# Integrative Reverse Genetic Analysis Identifies Polymorphisms Contributing to Decreased Antimicrobial Agent Susceptibility in *Streptococcus pyogenes*

**DOI:** 10.1128/mbio.03618-21

**Published:** 2022-01-18

**Authors:** Stephen B. Beres, Luchang Zhu, Layne Pruitt, Randall J. Olsen, Ahmad Faili, Samer Kayal, James M. Musser

**Affiliations:** a Laboratory of Molecular and Translational Human Infectious Disease Research, Center for Infectious Diseases, Department of Pathology and Genomic Medicine, Houston Methodist Research Institute and Houston Methodist Hospital, Houston, Texas, USA; b Departments of Pathology and Laboratory Medicine and Microbiology and Immunology, Weill Cornell Medical College, New York, New York, USA; c Inserm, CIC 1414, Rennes, France; d Université Rennes 1, Faculté de Pharmacie, Rennes, France; e CHU de Rennes, Service de Bacteriologie-Hygiène Hospitalière, Rennes, France; f Université Rennes 1, Faculté de Médecine, Rennes, France; MedImmune

**Keywords:** *Streptococcus pyogenes*, penicillin-binding proteins, molecular evolution, positive selection, antibiotic resistance, whole-genome sequencing, population genomics, β-lactams, beta-lactams

## Abstract

Identification of genetic polymorphisms causing increased antibiotic resistance in bacterial pathogens traditionally has proceeded from observed phenotype to defined mutant genotype. The availability of large collections of microbial genome sequences that lack antibiotic susceptibility metadata provides an important resource and opportunity to obtain new information about increased antimicrobial resistance by a reverse genotype-to-phenotype bioinformatic and experimental workflow. We analyzed 26,465 genome sequences of Streptococcus pyogenes, a human pathogen causing 700 million infections annually. The population genomic data identified amino acid changes in penicillin-binding proteins 1A, 1B, 2A, and 2X with signatures of evolution under positive selection as potential candidates for causing decreased susceptibility to β-lactam antibiotics. Construction and analysis of isogenic mutant strains containing individual amino acid replacements in penicillin-binding protein 2X (PBP2X) confirmed that the identified residues produced decreased susceptibility to penicillin. We also discovered the first chimeric PBP2X in S. pyogenes and show that strains containing it have significantly decreased β-lactam susceptibility. The novel integrative reverse genotype-to-phenotype strategy presented is broadly applicable to other pathogens and likely will lead to new knowledge about antimicrobial agent resistance, a massive public health problem worldwide.

## INTRODUCTION

Antibiotic resistance is of increasing concern in the treatment of microbial infections globally (1, 2). Streptococcus pyogenes (commonly known as group A Streptococcus [GAS]) is an important human pathogen that is among the top 10 infectious disease causes of morbidity and mortality, responsible for more than 700 million infections and 517 thousand deaths annually (3). Importantly, there is no licensed vaccine to prevent S. pyogenes infections (4). Penicillin and other β-lactam antibiotics that inhibit the synthesis of bacterial peptidoglycan (PG) are the primary agents used to treat S. pyogenes infections. High-molecular-mass penicillin-binding proteins (HMM PBPs) catalyze the polymerization of linear glycan strands (transglycosylation) and the cross-linking between strands (transpeptidation) to produce the PG sacculus, the major structural component of the bacterial cell wall. β-Lactam antibiotics bind covalently to the transpeptidase domain of the HMM PBPs and inhibit cross-linking.

A fortunate anomaly in S. pyogenes biology has been an enduring high susceptibility to β-lactam family antibiotics. To date, a naturally occurring penicillin resistant isolate has not been documented (5–7). This is unusual because β-lactam resistance has emerged multiple times independently in many other important Gram-positive human bacterial pathogens, including species of the genera Staphylococcus, *Enterococcus*, and Streptococcus (8). Following the introduction of penicillin in the early 1940s, resistance emerged within just a few years in staphylococci (9, 10) and enterococci (11), and soon thereafter resistant strains were described globally. In contrast, emergence of antimicrobial resistance in streptococci has been slower and subsequent global spread more gradual. Penicillin resistance in an important streptococcal human pathogen was not detected for another ∼25 years, when in the mid-1960s to 1970s there were sporadic reports of Streptococcus pneumoniae isolates of intermediate resistance in Australia, New Guinea, South Africa, and the United States (12–14). High-level penicillin resistance in pneumococci first emerged in the mid-1970s in South Africa (15) and then was described globally in the 1980s (14, 16). Identification of isolates with antibiotic resistance followed by subsequent genetic analysis resulted in the discovery that resistant strains have mosaic HMM PBPs not present in susceptible isolates (15, 17). This was followed ∼20 years later in the mid-1990s, by resistance emergence in Streptococcus agalactiae (also known as group B Streptococcus [GBS]) (18), and again ∼10 years later in 2007 in Canada (19). GBS clinical isolates with reduced β-lactam susceptibility are currently spreading in Japan (20) and the United States (21, 22). In 2010 to 2012, penicillin resistance was documented in four clonally related Streptococcus dysgalactiae subsp. *equisimilis* (SDSE) invasive isolates in Denmark (23). SDSE is the species most closely related to S. pyogenes and in some locations is the most prevalent cause of pyogenic streptococcal infections (24). For the pyogenic streptococcal species in which resistance has emerged, the evolution from penicillin susceptible to reduced susceptibility and then to nonsusceptible has occurred by incremental accumulation of amino acid substitutions in HMM PBPs and not by single-event horizontal gene transfer (HGT) of a β-lactamase or HMM PBP with low β-lactam affinity (8, 15, 18, 23, 25). Thus, identification of genetic polymorphisms in pathogenic streptococci that result in decreased antibiotic susceptibility has always proceeded from phenotypic observation to genotypic characterization. This phenotype-to-genotype work flow has dominated studies of the molecular basis of antibiotic resistance for many decades and has been the workhorse responsible for new mechanistic discoveries.

The potential for penicillin resistance under *in vivo* conditions has long been a concern in S. pyogenes pharyngitis infections. β-Lactam treatment failures have been documented over decades, and for reasons that are not fully understood, rates as high as 30 to 40% have been recorded in some case series (26, 27). Recently, interest in decreased penicillin susceptibility and potential for resistance emergence in S. pyogenes has been renewed by the report of two genetically related invasive isolates with 8-fold-higher MICs for ampicillin and amoxicillin and a 3-fold-higher MIC for cefotaxime (28). Both isolates had a mutation in *pbp2x*, producing a T_553_K substitution in a conserved PBP2X transpeptidation catalytic motif. Although the susceptibility level of these isolates does not exceed the *in vitro* resistance level defined for pyogenic beta-hemolytic streptococci, it was the first documentation of a naturally occurring PBP mutation in a S. pyogenes clinical isolate associated with reduced susceptibility *in vitro* to multiple β-lactam antibiotics. This finding prompted population genomic investigations of the diversity present in the HMM PBPs (29–31). These studies focused primarily on the transpeptidase domain of PBP2X and identified dozens of additional PBP2X amino acid substitutions among geographically widespread isolates (29–31). Several of these PBP2X substitutions were present in multiple isolates of diverse *emm* genetic lineages, indicating convergent evolution, and hypothesized to be the consequence of antibiotic treatment selecting for adaptations conferring reduced β-lactam susceptibility (30). Supporting this hypothesis, several PBP2X substitutions with these signatures of convergent evolution were associated with 2-to-3-fold increased MICs for one or more β-lactam antibiotics *in vitro* (30, 31). The ability of most of these substitutions to alter β-lactam susceptibility has not been proven experimentally. Thus far, isogenic mutant strains have been constructed only for two PBP2X substitutions, M_593_T and P_601_L, and each of these two amino acid changes were experimentally proven to be sufficient to increase the penicillin MIC 2- to 3-fold *in vitro* relative to wild-type PBP2X (30, 31). Moreover, the PBP2X P_601_L mutation also was shown sufficient to enhance survival *in vivo* under conditions of intermittent subtherapeutic antibiotic dosing in a mouse model of necrotizing fasciitis (32).

The continued susceptibility of S. pyogenes to β-lactams has led investigators to propose the existence of barriers such as DNases and restriction-modification systems, genetic constraints such as functional essentiality, or the lack of natural competence to explain the absence of β-lactam resistance in this organism (6, 29). Here, we used a novel integrative reverse genotype-to-phenotype strategy to identify polymorphisms that decrease susceptibility to β-lactam antibiotics. We determined the species-wide allelic diversity present in the four HMM PBPs (PBP1A, PBP1B, PBP2A, and PBP2X) by analysis of large global cohort of 26,465 S. pyogenes genomes. We demonstrate that the HMM PBPs have a significantly lower frequency of single nucleotide polymorphisms (SNPs) and recombination (i.e., are less genetically diverse) than the average core gene and are evolving primarily under purifying selection. Using multiple statistical tests and indicators of evolutionary selection, we identified specific HMM PBP amino acid substitutions with signals of positive selection hypothesized to confer decreased β-lactam susceptibility. We tested this hypothesis by constructing eight isogenic mutant strains with precisely defined amino acid replacements in PBP2X and show that these changes produce ∼2-fold increases in MICs for penicillin *in vitro*. Further, we discovered the first examples of interspecies horizontal acquisition by S. pyogenes of PBP2B and PBP2X likely from S. dysgalactiae donors and show that S. pyogenes strains with a naturally acquired chimeric PBP2X protein have significantly decreased susceptibility to several β-lactam antibiotics of the penicillin, cephalosporin, and carbapenem families. The novel integrative reverse genotype-to-phenotype strategy we used is broadly applicable to other pathogens and likely will result in new knowledge about antimicrobial agent resistance, a massive public health problem worldwide. Our findings are interpreted in the context of the potential for emergence of high-level β-lactam resistance in S. pyogenes.

## RESULTS

### Species-wide genomic genetic diversity.

**FIG 1:**
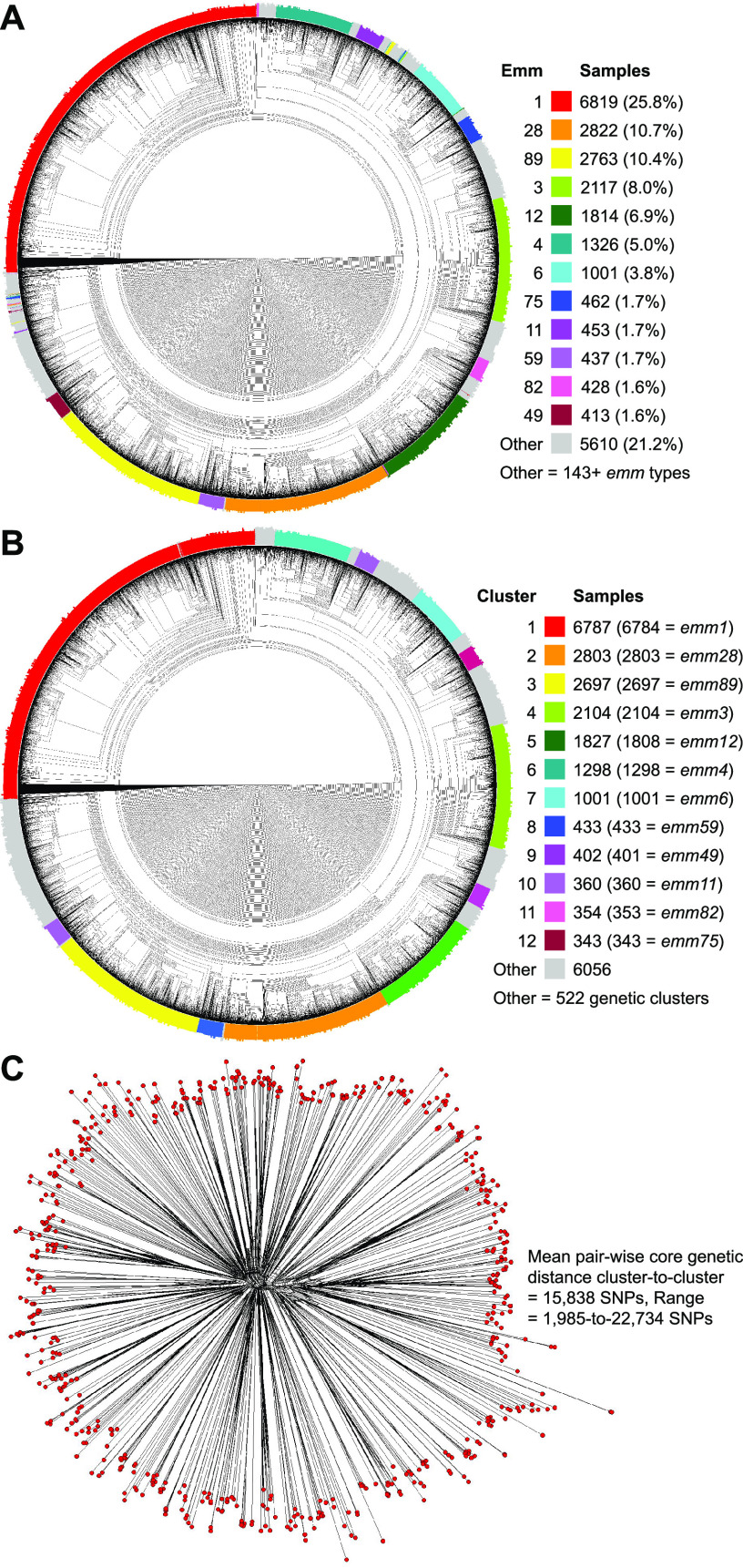
Genetic relationships among S. pyogenes genomes and genetic clusters. (A, B) Illustrated as a circular cladogram are genetic relationships inferred by neighbor-joining analysis among 26,465 S. pyogenes genome assemblies, based on 415,239 core chromosomal SNPs. Isolates are colored by the 12 most abundant *emm* types in panel A and by the 12 most abundant genetic clusters in panel B, as per the indices. Genetic clusters correspond closely with *emm* type (adjusted Rand index = 0.9873). (C) Radial phylogram illustrating genetic relationships among 595 isolates selected as representative of the genetic clusters encompassing the breadth of diversity in the cohort of 26,465 genomes. Phylogeny was inferred by neighbor-network analysis based on 283,697 core chromosomal SNPs. The starburst topology of the phylogeny is consistent with the cluster representatives being genetically distinct and similarly divergent from each other and a most recent common ancestor.

To evaluate the genetic diversity and evolution of the S. pyogenes four HMM PBPs (1A, 1B, 2A, and 2X) species-wide, publicly available whole-genome sequencing (WGS) data were obtained for 26,465 samples (see Table S1 in the supplemental material). The 26,465 genome sequences are of 157+ *emm* types (“+” indicates some samples were nontypeable since they were variants not present in *emm* typing databases) and 673+ multilocus sequence types (MLSTs) (Fig. 1A). The 12 most abundant *emm* types (1, 28, 89, 3, 12, 4, 6, 75, 11, 59, 82, and 49 [in descending order]) account for 78.8% (*n *=* *20,855) of the cohort, and the remaining 21.2% (*n *=* *5,610) are of 146+ *emm* types.

10.1128/mbio.03618-21.3 TABLE S1 Cohort of 26,465 S. pyogenes whole-genome sequencing data sets. Download Table S1, XLSX file, 3.1 MB. Copyright © 2022 Beres et al. 2022 Beres et al. https://creativecommons.org/licenses/by/4.0/ This content is distributed under the terms of the Creative Commons Attribution 4.0 International license.

To establish a genetic framework within which the evolution of the HMM PBPs could be interpreted, the population structure of the cohort was evaluated by density-based spatial clustering (DBSCAN) for lineages of core genomes significantly more closely genetically related to each other than at random. The 26,465 genomes were clustered into 534 genetic lineages (Fig. 1B; see also Table S1 and Fig. S1). These lineages correlated well with *emm* type and MLST (adjusted Rand indices = 0.9873 and 0.8056, respectively). Based on this clustering a set of 595 core genomes representative of the population structure were selected (Fig. 1C; see also Table S1). These 595 genomes are of genetic lineages encompassing 26,414 (99.8%) isolates of the cohort. The phylogenetic network inferred for these 595 core genomes has a starburst topology consistent with the individual isolates being similarly divergent from each other (Fig. 1C). The isolates from lineage to lineage differed on average by 15,838 core SNPs (range = 1,985 to 22,734), a value consistent with between *emm* types.

10.1128/mbio.03618-21.1 FIG S1 Determination of S. pyogenes genetic lineages. Initial (A) and refined (B) model fits for hierarchical density-based spatial clustering of 26,465 S. pyogenes pseudo core genomes. Pseudo core genomes were generated based on 415,239 core SNPs identified relative to the genome of reference M89 strain MGAS23530. Population partitions were determined using PopPUNK v2.3.0. Download FIG S1, PDF file, 0.6 MB. Copyright © 2022 Beres et al. 2022 Beres et al. https://creativecommons.org/licenses/by/4.0/ This content is distributed under the terms of the Creative Commons Attribution 4.0 International license.

### HMM PBP diversity and genetic relationships.

**FIG 2:**
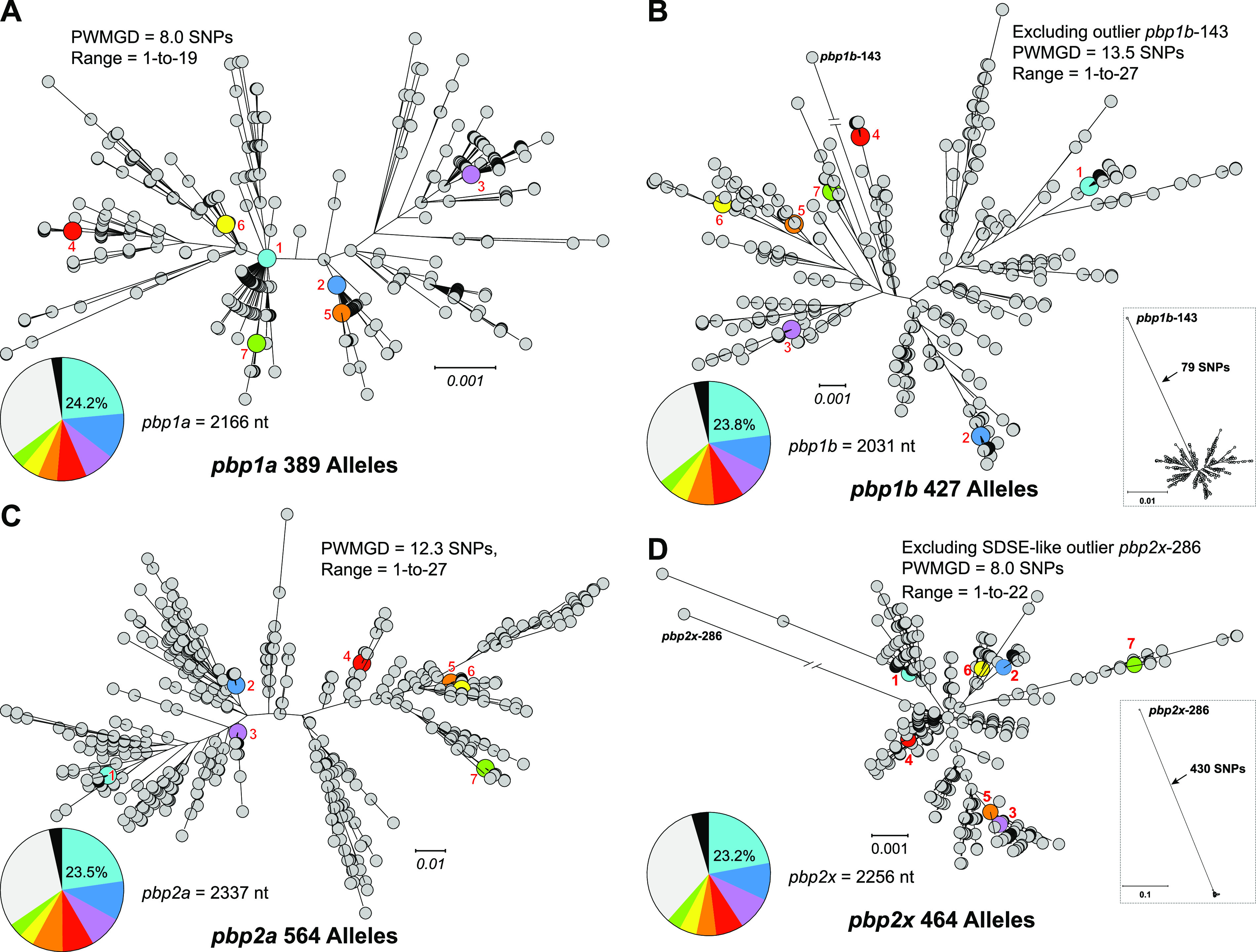
Genetic relationships among HMM PBP alleles. Relationships among alleles of each of the four HMM PBP genes were inferred by maximum likelihood and are illustrated as radial phylograms: *pbp1a* (A), *pbp1b* (B), *pbp2a* (C), and *pbp2x* (D). The pairwise mean genetic distance (PWMGD) in terms SNP differences between alleles is provided for each allele set. The inset trees (lower right) in panels B and D show the greater genetic distance of the recombinant outlier alleles *pbp1b*-143 and *pbp2x*-286 relative to the more closely related alleles of PBP1B and PBP2X, respectively. The seven most prevalent alleles for each of the PBPs are numbered and indicated in color. The inset pie charts (lower left) illustrate the relative abundance of the seven most prevalent alleles (gray = other alleles, black = undetermined). Among the isolates for which an allele was determined, the seven most prevalent alleles account for 66.7% (17,155/25,721) of *pbp1a* sequences, 66.9% (17,002/25,410) of *pbp1b* sequences, 67.8% (17,323/25,547) of *pbp2a* sequences, and 64.4% (16,281/25,265) of *pbp2x* sequences. In contrast, 30.6% (119/389) of *pbp1a* alleles were present in only a single sample in the strain cohort versus 30.4% (130/427) for *pbp1b*, 41.3% (233/564) for *pbp2a*, and 37.8% (175/464) for *pbp2x*.

**FIG 3:**
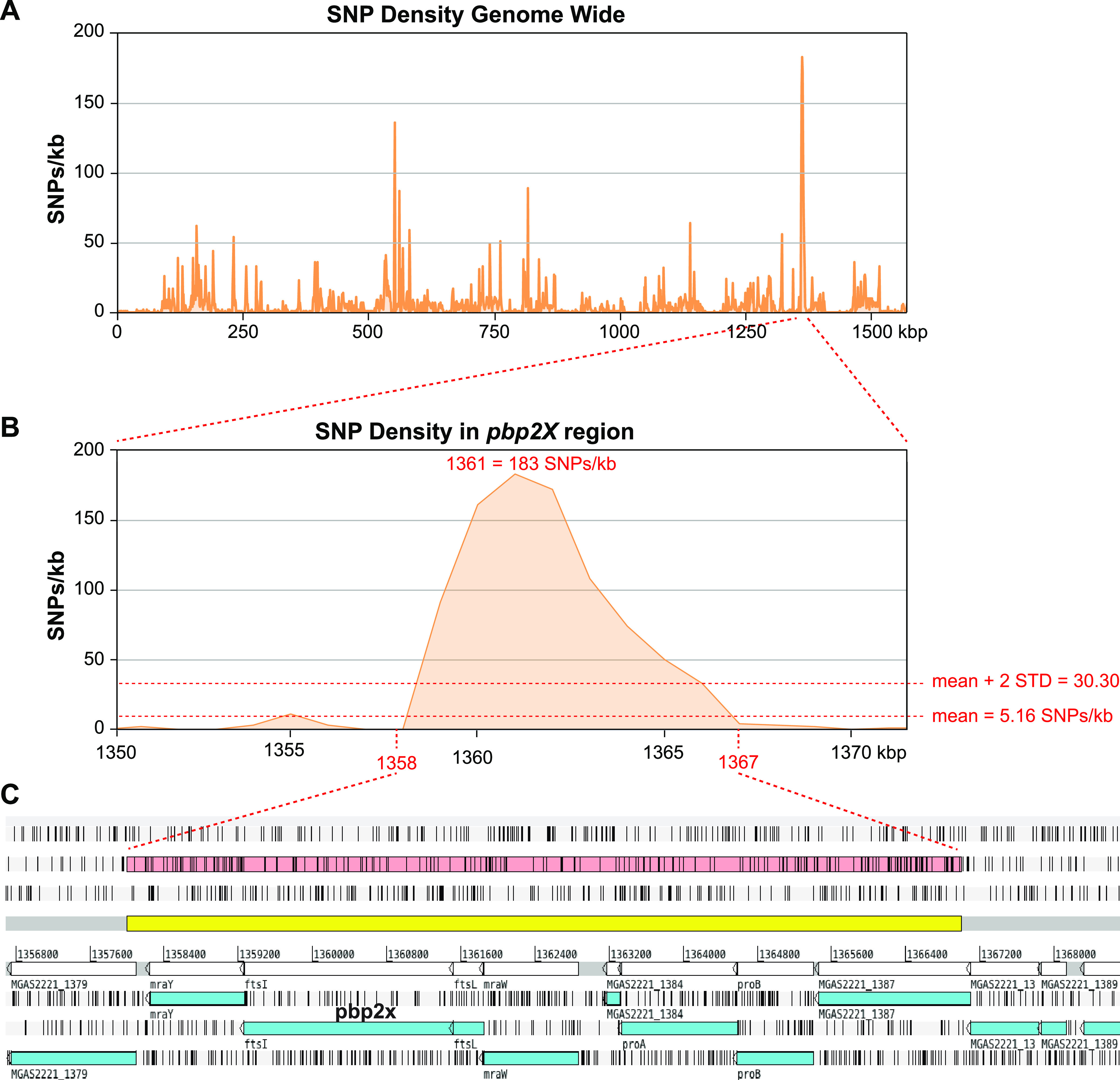
Acquisition of an SDSE-like *pbp2x* gene by horizontal gene transfer. (A and B) Genome-wide density of SNPs identified in sample ERR2650320 (aka SAME3198851) relative to Emm1 reference strain MGAS2221 (A) and in the region surrounding *pbp2x* (B). SNP density is greatest in the region encompassing *pbp2x* and is significantly greater than the genome average. (C) Gene content in the *pbp2x* region of elevated SNP density. The region highlighted in yellow indicates the ∼9-kb recombinant segment with an elevated SNP density. The seven genes: *proB*-*proA*-(MGAS2221_1384)-*mraW*-*ftsL*-*pbp2x*-*mraY* have 100% nucleotide identity with the cognate genes of SDSE strain NCTC11554, consistent with HGT acquisition from an SDSE donor. SDSE, Streptococcus dysgalactiae subsp. *equisimilis*.

We identified 389 *pbp1a* alleles, 427 *pbp1b* alleles, 564 *pbp2a* alleles, and 464 *pbp2x* alleles of (see Table S2) among the 26,465 genome assemblies by BLAST search. For each of the PBPs, a full-length allele was obtained from over 95% of the WGS assemblies (see Table S3). Sequence conservation was relatively higher for the three essential PBPs (33–35) (PBP1A, PBP1B, and PBP2X), where ∼85% of nucleotide (nt) sites were invariant, than for dispensable PBP2A, where ∼83% of nucleotide sites were invariant (see Table S4). As expected, the most prevalent alleles occurred in the most prevalent genetic lineages/*emm* types. There was no single allele of any of the HMM PBPs that accounted for a majority of the isolates (Fig. 2). The seven most prevalent alleles (each present in greater than 1,000 isolates) accounted for 64 to 68% of the sequences for each of the PBP genes in the cohort. Conversely, for each PBP, 30 to 40% of the alleles were unique, being identified in only a single isolate of the strain cohort. All *pbp1a* and *pbp2a* alleles were closely related, sharing 99.6 and 99.5% average nucleotide identities (ANI), respectively (Fig. 2A and C). Similarly, for *pbp1b* and *pbp2x* nearly all alleles were closely related (Fig. 2B and D). However, for each of the *pbp1b* and *pbp2x* allele sets, one genetic outlier was identified, alleles *pbp1b*-427 and *pbp2x*-286, respectively (Fig. 2B and D, insets). Excluding these genetic outliers, the alleles of *pbp1b* and *pbp2x* share 99.4 and 99.6% ANI, respectively. Outlier alleles *pbp1b*-427 and *pbp2x*-286 differ from the other 426 *pbp1b* alleles and the other 463 *pbp2x* alleles by 79 (96.6% ANI) and 430 (80.9% ANI) SNPs, respectively. Outlier allele *pbp1b-*427 has a recombinant mosaic structure, with an internal segment of 345 nt (i.e., nt 816 to 1160 of the 2,301 bp of *pbp1b* of strain MGAS2221) being divergent from the other *pbp1b* alleles. This segment is identical to the *pbp1b* sequence of SDSE strain RE378. Recombinant allele *pbp1b*-143 was present in four isolates of type *emm65*, three collected in Kenya between 2012 to 2015 and one collected in the United States in 2017, indicating the geographic spread of this mosaic *pbp1b* allele. Similarly, outlier allele *pbp2x-*286 is divergent over its entire 2,256 bp relative to the other *pbp2x* alleles but is identical to *pbp2x* sequence of SDSE strain NCTC11554. Horizontally acquired allele *pbp2x*-286 was present in one isolate of *emm238* collected in New Zealand in 2012. Analysis of the genome of the isolate from which *pbp2x*-286 was obtained (ERR2650320; aka SAMEA3918851) shows an SDSE-like region of recombination to be 9 kbp in length and encompass 7 additional genes flanking *pbp2x* (Fig. 3). Included among these flanking genes directly adjacent to *pbp2x* and part of the same transcriptional unit are 5′ *mraY*, 3′ *ftsL*, and *mraW* (36). The four genes in this transcriptional unit (*mraY-pbp2x-ftsL-mraW*) each encode a product involved in peptidoglycan synthesis and/or cell division.

10.1128/mbio.03618-21.4 TABLE S2 HMM PBP alleles and variants. Download Table S2, XLSX file, 0.4 MB. Copyright © 2022 Beres et al. 2022 Beres et al. https://creativecommons.org/licenses/by/4.0/ This content is distributed under the terms of the Creative Commons Attribution 4.0 International license.

10.1128/mbio.03618-21.5 TABLE S3 HMM PBP gene statistics. Download Table S3, DOCX file, 0.01 MB. Copyright © 2022 Beres et al. 2022 Beres et al. https://creativecommons.org/licenses/by/4.0/ This content is distributed under the terms of the Creative Commons Attribution 4.0 International license.

10.1128/mbio.03618-21.6 TABLE S4 HMM PBP gene nucleotide variation statistics. Download Table S4, DOCX file, 0.01 MB. Copyright © 2022 Beres et al. 2022 Beres et al. https://creativecommons.org/licenses/by/4.0/ This content is distributed under the terms of the Creative Commons Attribution 4.0 International license.

**FIG 4:**
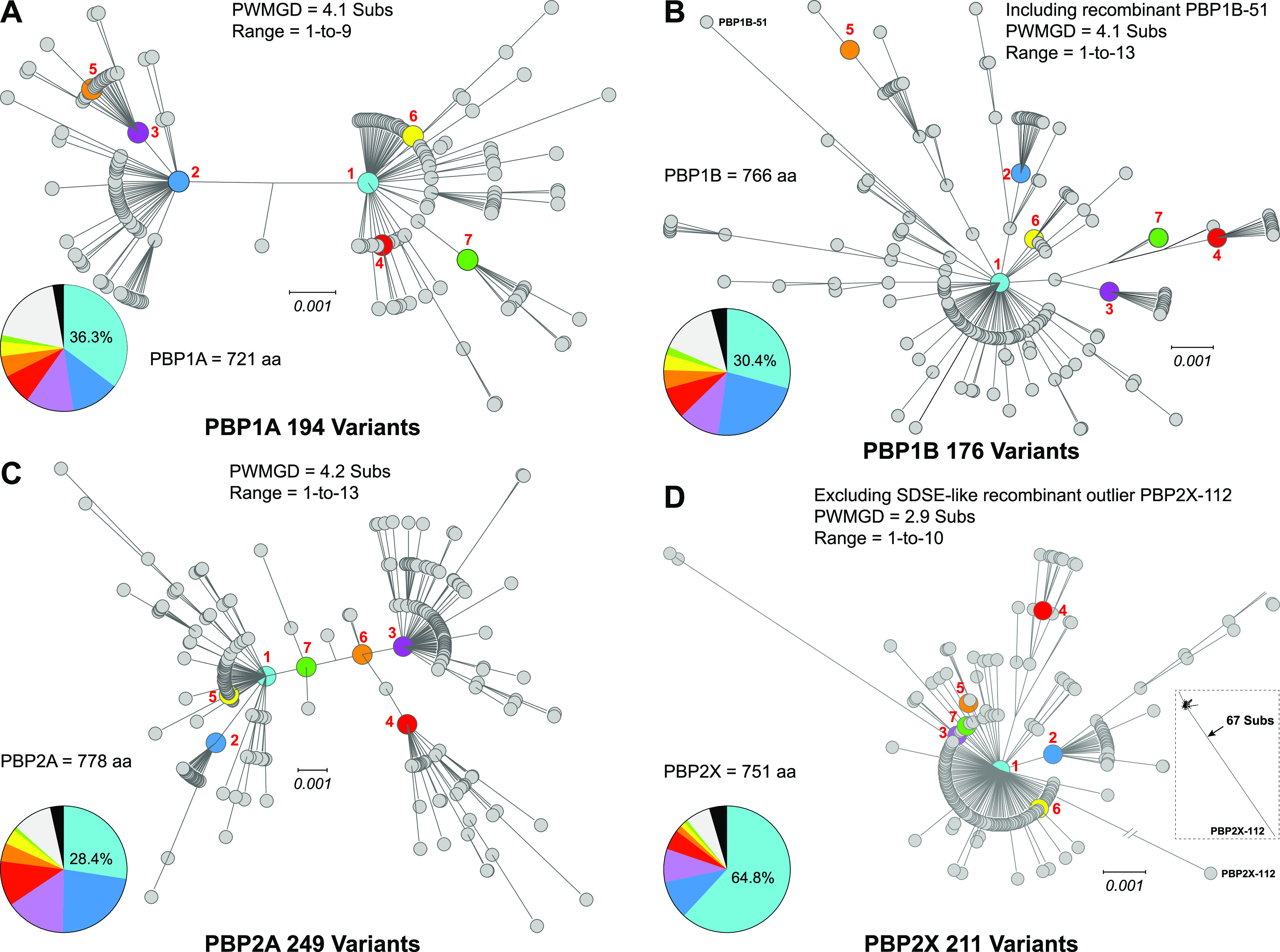
Genetic relationships among HMM PBP variants. Genetic relationships among the protein variants of each of the four HMM PBP gene alleles were inferred by maximum likelihood and are illustrated as radial phylograms: PBP1A (A), PBP1B (B), PBP2A (C), and PBP2X (D). Variant PBP1B-51 in panel B is the translated product of genetic outlier recombinant allele *pbp1b*-143 and differs from the most prevalent PBP1B variant by seven amino acid substitutions. The inset tree (lower right) in panel D shows the greater genetic distance of the recombinant outlier variant PBP2X-112, the translated product of SDSE-like outlier allele *pbp2x*-286, relative to the more closely related variants of PBP2X. The seven most prevalent variants of each of the PBPs are numbered and indicated in color. The inset pie charts in each panel (lower left) illustrate the relative abundance of the seven most prevalent variants (gray = other variants, black = undetermined). Among the isolates for which a variant was determined, the seven most prevalent variants account for 80.6% (20,736/25,721) of PBP1A sequences, 84.9% (21,572/25,410) of PBP1B sequences, 89.7% (22,910/25,547) of PBP2A sequences, and 93.4% (23,593/25,265) of PBP2X sequences. In contrast, 33.5% (65/194) of PBP1B variants were present in only a single sample in the strain cohort versus 35.8% (63/176) for PBP1B, 42.6% (106/249) for PBP2A, and 44.6% (94/211) for PBP2X.

Translation of the PBP gene alleles produced 194 variants of PBP1A, 176 of PBP1B, 249 of PBP2A, and 211 of PBP2X. With the exception of the horizontally acquired SDSE-like variant PBP2X-112, for each of the PBPs the variants are closely related, having greater than 99.4% average amino-acid identity (AAI) (Fig. 4). Although there are 67 nt changes in the 345-bp SDSE-like recombination block that differentiates outlier allele *pbp1b*-143 from the most prevalent *pbp1b* allele (Fig. 2B, inset), only seven of these SNPs are nonsynonymous and result in amino acid substitutions that differentiate recombinant variant PBP1B-51 from PBP1B-1, the most prevalent PBP1B variant (Fig. 4B).

In general, the most prevalent variants of PBP1A, PBP1B, and PBP2A correspond to the most prevalent genetic lineages of the cohort. For these three class A PBPs, no single protein variant accounted for more than 23 to 36% of the cohort isolates (Fig. 4A to C). In contrast, there is less diversity among the class B PBP2X variants relative to the other PBPs (Fig. 4D). Among the PBP2X variants, PBP2X-1 accounts for 64.8% of the cohort isolates. The PBP2X-1 variant is encoded by 33.2% of the *pbp2x* alleles and was identified in one or more isolates of ∼2/3 (335/534 = 62.7%) of the cohort genetic lineages. In addition, another 63.0% of the PBP2X variants differ from PBP2X-1 by only a single amino acid substitution. The lower diversity among the PBP2X variants relative to the other PBPs and the predominance of the PBP2X-1 variant, is consistent with strong convergent purifying selection acting on PBP2X.

### HMM PBP evolutionary selection.

The lack of β-lactam resistance in S. pyogenes has been posited to be due in part to constraints on molecular processes that permit or promote genetic diversification (6, 29). Given that the cell wall is vital to the structural integrity of bacteria, it is reasonable to speculate that diversification of the PBPs is functionally constrained by the essential activity these enzymes have in PG synthesis and maintenance. However, formal analysis of the evolutionary selective forces acting on the PBPs to test this hypothesis and address the nature of potential constraints has not been done.

As an initial assessment of the evolutionary forces acting on the PBPs and to place them in context relative to the other S. pyogenes genes, the nature and distribution of SNPs among 1,572 S. pyogenes core genes was examined. Among the 26,456 WGS assemblies (WGS samples with exogenous SDSE-like blocks of recombination within the PBPs were excluded because of their potential to distort inferences of evolutionary selection), 456,441 SNPs were identified at 413,659 polymorphic sites across the core genome, of which 374,427 (82.0%) SNPs occurred in coding sequences (CDS) at 341,926 polymorphic sites. Polymorphic sites accounted for 24.0% (413,659/1,723,492) of the core genome, with a SNP occurring on average every 4.2 bp. The average among the 1,572 core genes was one SNP site every 3.9 bp or ∼255 SNPs per kb of CDS.

Among the CDS SNPs, 44.3% were synonymous (166,038 sSNPs) and 55.7% were nonsynonymous (208,389 nsSNPs, including nonsense mutations). In comparison, all four of the HMM PBP genes had a significantly lower frequency of SNPs than the average gene, with SNPs being 27.3-to-36.1% less prevalent (Table 1; see also Table S5). The lower frequency was most pronounced for nsSNPs, that were 37.1 to 47.9% less prevalent. Although sSNPs among the PBPs were less frequent than expected for a random distribution, this reduction was not statistically significant for *pbp1a* and *pbp2x*. The less pronounced reduction in sSNPs relative to the nsSNPs among the PBPs, is consistent with the sSNPs being largely selectively neutral. The overall reduction in SNPs, and specifically nsSNPs, is consistent with the PBPs evolving largely under negative/purifying selection.

**TABLE 1:** SNP frequencies in HMM PBP genes and the average core gene

Gene	Length (nt)	SNP frequency^*a*^						
		SNPs	sSNPs	nsSNPs	SNPs/kb	sSNPs/kb	nsSNPs/kb	nsSNP/sSNP
Avg	932.0	238.2	105.6	132.6	255.3	109.3	146.0	1.445
*pbp1a*	2,166	357	185	172	164.8*	85.4*	79.4*	0.930
*pbp1b*	2,301	389	214	175	169.1*	93.0	76.1*	0.818
*pbp2a*	2,337	434	216	218	185.7*	92.4	93.3*	1.009
*pbp2x*	2,256	368	177	191	163.1*	78.5*	84.7*	1.079

a *, frequencies that differ significantly from the average.

10.1128/mbio.03618-21.7 TABLE S5 HMM PBP gene SNP distribution with respect to the average core gene. Download Table S5, DOCX file, 0.01 MB. Copyright © 2022 Beres et al. 2022 Beres et al. https://creativecommons.org/licenses/by/4.0/ This content is distributed under the terms of the Creative Commons Attribution 4.0 International license.

We next examined the distribution of the SNPs with respect to the transglycosylase (TG) and transpeptidase (TP) domains (see Tables S6 and S7). That is, we investigated if the lower than random frequency of SNPs was specific to a functional domain/catalytic activity. Total SNPs and sSNPs were not significantly nonrandomly distributed with respect to either the TG or TP domains for any of the PBPs. Similarly, for the TP domain the frequency of nsSNPs deviated by 10% or less from expectation for a random distribution. In contrast, for the TG domain of each of the three class A PBPs (*1a*, *1b*, and *2a*), nsSNPs were ∼30% less frequent than expected at random; however, this difference was not significant. The consistently lower abundance of nsSNPs in the TG domain compared to the TP domain suggests that a requirement for maintenance of TG activity may impart greater purifying constraints than TP activity. Alternatively, given that the TP domain is the site of β-lactam binding, this finding may reflect β-lactam antibiotics exerting positive/diversifying adaptive selection on the TP domain but not on the TG domain.

10.1128/mbio.03618-21.8 TABLE S6 HMM PBP transglycosylase domain SNP distribution. Download Table S6, DOCX file, 0.01 MB. Copyright © 2022 Beres et al. 2022 Beres et al. https://creativecommons.org/licenses/by/4.0/ This content is distributed under the terms of the Creative Commons Attribution 4.0 International license.

10.1128/mbio.03618-21.9 TABLE S7 HMM PBP transpeptidase domain SNP distribution. Download Table S7, DOCX file, 0.01 MB. Copyright © 2022 Beres et al. 2022 Beres et al. https://creativecommons.org/licenses/by/4.0/ This content is distributed under the terms of the Creative Commons Attribution 4.0 International license.

**FIG 5:**
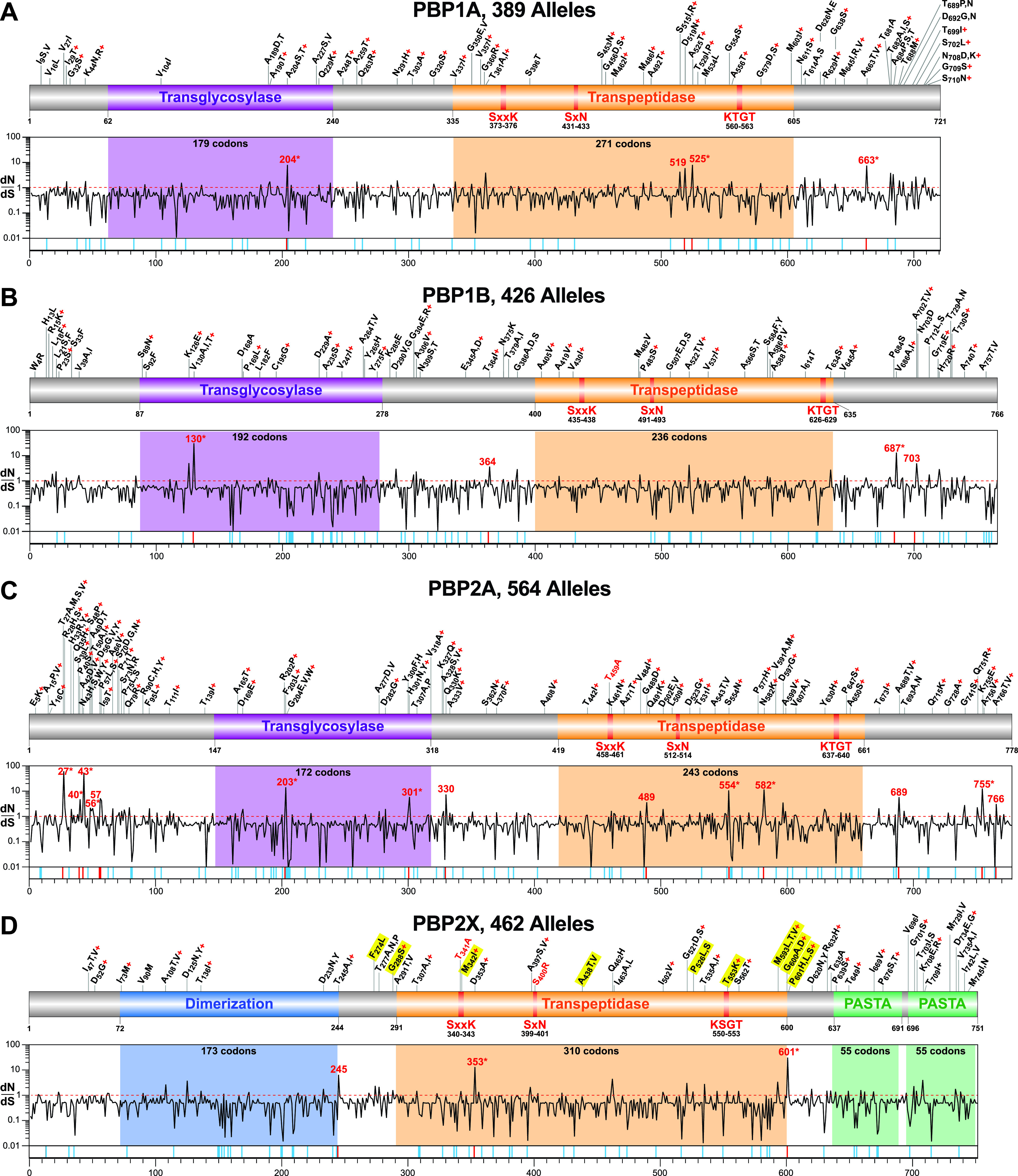
HMM PBP amino acid substitution sites and inferred signatures of evolutionary selection. (A to D) Schematics of the HMM PBPs are illustrated at the top of each panel. Shown are the functional domains and sites of parallel/convergent substitutions identified relative to the most prevalent variant. Substituted sites identified in two or more genetic lineages are indicated in black, and sites found in a single lineage but located within one of the conserved catalytic motifs of the transpeptidase domain are indicated in red. Sites identified as homoplasic are indicated by a plus sign in red. PBP2X substitutions linked with reduced susceptibility to one or more β-lactam antibiotics *in vitro* are highlighted in yellow. Graphed below each PBP schematic is the *dN*/*dS* ratio for each individual codon site determined with FUBAR. Sites identified as having a significant probability of evolving under positive selection by at least one test method (FUBAR, MEME, or SLAC) are numbered in red, and sites identified as significant by multiple methods are indicated by an asterisk. Indicated with tic marks below the graphs are significantly negatively selected sites in blue and significantly positively selected sites in red. To simplify the figures and because of their potential to distort evolutionary inferences, PBP genetic outlier recombinant alleles/variants were excluded from the generation of the schematics and the *dN*/*dS* determinations.

The primary process by which streptococcal species have evolved decreased β-lactam susceptibility is by accumulation of amino acid substitutions in their native PBPs that reduce antibiotic binding affinity (8). Such substitutions can arise by spontaneous point mutations or by homologous recombination. Antibiotic treatment can provide an environment strongly selective for substitutions conferring reduced susceptibility and result in a limited set of high fitness adaptations repeatedly occurring in different genetic lineages of an organism by convergent positive selection (37, 38). A molecular signal of such parallel or convergent evolution is SNPs that are homoplasic (i.e., evolutionarily inconsistent with the clonal frame) within the context of the population structure (39). Therefore, to determine whether there were signals of fitness selection and adaptive evolution, the PBP sequences were examined for substitutions present in multiple divergent genetic lineages and for SNPs homoplasic at the codon level. Among the *pbp1a* alleles, 56 convergent codon sites were identified that had one or more substitutions present in two or more genetic lineages, 58 for *pbp1b*, 76 for *pbp2a*, and 46 for *pbp2x* (Table 2 and Fig. 5). For each of the PBPs there was substantial overlap in the codon sites identified as convergent with sites identified as homoplasic (53 to 95%). Apart from an overabundance of sites identified at the amino terminal end of PBP2A (Fig. 5C), there was no apparent nonrandom pattern to the distribution of the convergent and/or homoplasic sites across the PBP sequences. Of note, although PBP2X did not have the lowest number of substituted codon sites, it did have both the lowest number and the lowest proportion of either convergent or homoplasic sites. This finding is consistent with the lower diversity present among the PBP2X variants (Fig. 4) and might reflect stronger negative/purifying selection acting on PBP2X than on the other HMM PBPs.

**TABLE 2:** HMM PBP convergent and homoplasic codon sites^*a*^

PBP	No. of codon site(s) (%)			No. of convergent and homoplasic sites
	Substituted (assemblies, alleles)	Convergent (assembly based)	Homoplasic (allele based)	
1A	148, 141	56/148 (37.8)	43/141 (30.5)	41
1B	156, 154	58/1567 (37.2)	37/154 (24.0)	31
2A	185, 175	76/185 (41.1)	86/175 (49.1)	66
2X	161, 168	44/161 (27.3)	35/168 (20.8)	28

a Convergent codon sites (sites with amino acid substitutions identified in multiple genetic lineages), were determined by correlating SNPs derived from comparing the 26,456 WGS assemblies with the genetic lineages defined by HDBSCAN. Homoplasic codon sites were determined among 1,248 unique concatenated PBP alleles using SNPPar. The different input sets evaluated, and methods used are responsible for the modest difference in total substituted codon sites identified. The recombinant genetic outliers (*pbp1b*-143 and *pbp2x*-286) with SDSE-like sequences were excluded from both analyses.

As an additional test of fitness adaptation, we evaluated PBP allele phylogenies for sites evolving non-neutrally using three different site-by-site codon substitution models of episodic or pervasive selection: FUBAR, MEME, and SLAC (40–42). The rate of nonsynonymous mutations at nonsynonymous sites (*dN*) and the rate of synonymous mutations at synonymous sites (*dS*) was calculated for all codon alignment sites of the PBP genes, to infer sites evolving under positive (*dN*/*dS* > 1), neutral (*dN*/*dS* = 1), or negative (*dN*/*dS* < 1) selection (Table 3 and Fig. 5). Consistent with SNP data indicating purifying selection, a majority of codon sites (88.8 to 93.1%, percentages excluding the *pbp1b-*143 and *pbp2x-*286 recombinant outliers) for each of the PBPs have a *dN*/*dS* ratio less than 1, and a minority of sites (6.9 to 14.9%) have a ratio greater than 1. Most sites were deemed selectively neutral, since only 6.3 to 11.8% of sites with a *dN*/*dS* less than 1 were identified as evolving under statistically significant negative selection, and only a small number of sites with a *dN*/*dS* ratio greater than 1 as under significant positive selection. For the essential PBPs, 1A, 1B, and 2X (33, 34), there were only 3 to 4 sites per each determined to be under significant positive selection, whereas the dispensable PBP2A had 14 sites (Fig. 5). There was no apparent simple relationship between the sites evolving under either positive or negative selection with the functional domains of the PBPs. That is, with the exception PBP2A that has several positively selected sites clustered near the amino terminus, sites evolving non-neutrally appeared to be randomly distributed across the PBP sequences.

**TABLE 3:** HMM PBP codons sites evolving under nonneutral selection

PBP allele set^*a*^	Selection^*b*^	No. (%) of sites	Significant sites			Combined		
			FUBAR	MEME	SLAC	Gene-wide	TG/DM	TP
*pbp1a*-389	Neg	671 (91.4)	42	–	23	42	9	17
	Pos	63 (8.6)	4	3	0	4	1	2
*pbp1b*-426	Neg	682 (88.8)	66	–	49	67	20	14
	Pos	86 (11.2)	3	3	2	4	1	0
*pbp1b*-427*	Neg	714 (93.0)	75	–	55	76	22	17
	Pos	54 (7.0)	2	5	2	5	1	1
*pbp2a*-564	Neg	663 (85.1)	70	–	60	78	24	24
	Pos	116 (14.9)	12	9	5	14	2	3
*pbp2x*-462	Neg	700 (93.1)	53	–	36	53	17	24
	Pos	52 (6.9)	3	1	2	3	0	1
*pbp2x*-463*	Neg	733 (97.6)	299	–	53	299	75	132
	Pos	18 (2.4)	4	16	1	18	3	6

a *, allele sets include recombinant outliers (*pbp1b*-143 and *pbp2x*-286) with exogenous SDSE-like sequences.

b Selection as determined with FUBAR: negative = *dN/dS* < 1, positive = *dN/dS* > 1. Neg, negative; Pos, positive.

### PBP2X substituted codon sites and decreased β-lactam susceptibility.

One goal of this investigation is the identification of naturally occurring amino acid substitutions that may decrease the binding affinity of HMM PBPs for β-lactams and contribute to decreased antibiotic susceptibility. Identification of such sites can aid our understanding of the evolution of resistance and structure-function relationships between enzyme and antibiotic. Unlike many bacterial pathogens, β-lactam susceptibility testing is not routinely performed on S. pyogenes; thus, there is a paucity of antibiotic susceptibility information available to analyze. An association between specific individual amino acid changes in the S. pyogenes HMM PBPs and altered β-lactam susceptibility has been reported for substitutions at 29 sites of PBP2X in strains of five *emm* types (1, 12, 28, 43, and 89) (28, 30, 31). Isolates with substitutions at these sites were tested for altered β-lactam susceptibility, in part because the changes were located in a transpeptidase catalytic motif and present in multiple samples and/or in two or more *emm* types, suggesting positive selection and convergent evolution (28, 30, 31). An association with increased MICs to one or more β-lactams *in vitro* was found for 11 of the 29 sites (28, 30, 31). However, the capacity to cause reduced β-lactam susceptibility has been experimentally tested with isogenic mutant constructs for only two PBP2X substitutions, M_593_T and P_601_L (30, 31). Therefore, working under the hypothesis that β-lactam therapy is a primary selective force acting on the HMM PBPs, we examined the relationship between codon sites with signals of positive selection and substitutions at the 11 sites either associated with or experimentally proven to confer decreased susceptibility *in vitro* to one or more β-lactams (37) (Table 4 and Fig. 5D). Of 11 substituted sites, 10 are located either within or closely flank the PBP2X TP domain (Fig. 5D). For the cohort studied here among these 11 sites, 8 have a *dN*/*dS* ratio greater than 1, 9 were found in two or more genetic lineages, 6 were homoplasic, and 6 have all three of these signals of evolving under positive selection (Table 4). Although 8 of the 11 sites had *dN*/*dS* data indicating positive selection, it was statistically significant only for codon site 601. This suggests that in this analysis, independent of statistical significance, a *dN*/*dS* ratio greater than 1 is a reasonable predictor (at least for substitutions in the PBP2X transpeptidase domain) of the potential for conferring decreased β-lactam susceptibility. There are 10 additional untested sites within the TP domain of PBP2X that have one or more of these signals, 20 within the TP domain of PBP1A, 14 in PBP1B, and 22 in PBP2A (Fig. 5A to C). In addition, there are two untested PBP2X sites, T341A and S400R, that are located in TP conserved catalytic motifs (Fig. 5D in red). It should also be noted that previously tested sites with signals of positive selection that were not associated with reduced β-lactam susceptibility (18 of 29), may have reduced susceptibility to β-lactam antibiotics that were not tested or to the tested antibiotics but under different conditions, such as *in vivo*. The level of expression of the PBPs *in vivo* and at different anatomic sites of infection may be substantially different than *in vitro*. Except for the P_601_L substitution in PBP2X (32), an adaptation that enhances survival under laboratory conditions designed to mimic suboptimal β-lactam antibiotic therapy in a mouse model of necrotizing fasciitis, nothing is known about the consequences of any of these mutations on pathogenesis or fitness during human or experimental animal infection.

**TABLE 4:** Correlation between PBP2X codon sites with signatures of positive selection and altered β-lactam susceptibility

Site	Substitution(s)^*a*^	*dN/dS*	Bayes (*dS* < *dN*)	Lineage(s)	Homplasic	MIC pen G^*b*^	MIC change
274	Phe→Leu	2.329	5.272	2	No	–	–
288	Gly→Ser*	1.568	3.843	2	Yes	0.032	2.00
342	Met→Ile*	1.441	6.309	6	Yes	0.023	1.44
425	Phe→Leu	0.911	2.117	1	No	–	–
438	Ala→Thr	0.887	2.027	2	No	–	–
	Ala→Val			1		–	–
461	Thr→Pro	1.343	2.751	1	No	–	–
526	Pro→Leu	1.386	3.547	1	No	–	–
553	Thr→Lys*	1.474	3.698	2	Yes	0.023	1.44
593	Met→Leu*	3.398	12.301	2	Yes	0.032	2.00
	Met→Thr			6		0.032	2.00
	Met→Val*			6		0.032	2.00
600	Gly→Asp*	0.762	0.519	6	Yes	0.023	1.44
601	Pro→His*	34.421	12123	3	Yes	0.032	2.00
	Pro→Leu			10		0.032	2.00
	Pro→Ser*			1		0.016	1.00

a *, new isogenic strains constructed in this study; M_593_T and P_601_L mutants were described previously (30, 31).

b MICs provided for 10 PBP2X isogenic constructs with the fold change relative to the parent strain MGAS27213_L_601_P producing wild-type variant PBP2X-1 (penicillin G MIC = 0.016 μg/mL).

To directly test the hypothesis that PBP2X codon sites with signals of positive selection mediate decreased penicillin susceptibility *in vitro*, we constructed and analyzed isogenic mutant strains for eight additional substitutions at six codon sites with such signals (Table 4). WGS showed that the isogenic mutant strains lacked spurious mutations that might influence antibiotic susceptibility. With only one exception, the isogenic mutant strains with the single substituted amino acid were less susceptible to penicillin G *in vitro* (Table 4). These results show that the integrated population genomic analysis strategy we used can effectively identify candidate mutations that alter β-lactam susceptibility.

### HMM PBPs and HGT/recombination.

HGT and homologous recombination is a second molecular process that has contributed to HMM PBP diversifying evolution and the emergence of β-lactam resistance in other pathogenic bacteria (8). For example, high level β-lactam-resistant S. pneumoniae isolates have one or more mosaic HMM PBP genes whose products have reduced β-lactam binding affinity (43, 44). The mosaic structure of these PBP genes is attributed to interspecies recombination events mediated by transformation of naturally competent S. pneumoniae with exogenous DNA from commensal oral streptococcal donor species, predominantly S. mitis and S. oralis (45, 46). Given that S. pyogenes is not naturally competent, and mosaic PBP genes (excluding the outlier alleles identified in this investigation) have not been found, it has been proposed that the lack of β-lactam resistance in the organism is attributable to barriers to or inefficient mechanisms for HGT and recombination (6, 29).

**FIG 6:**
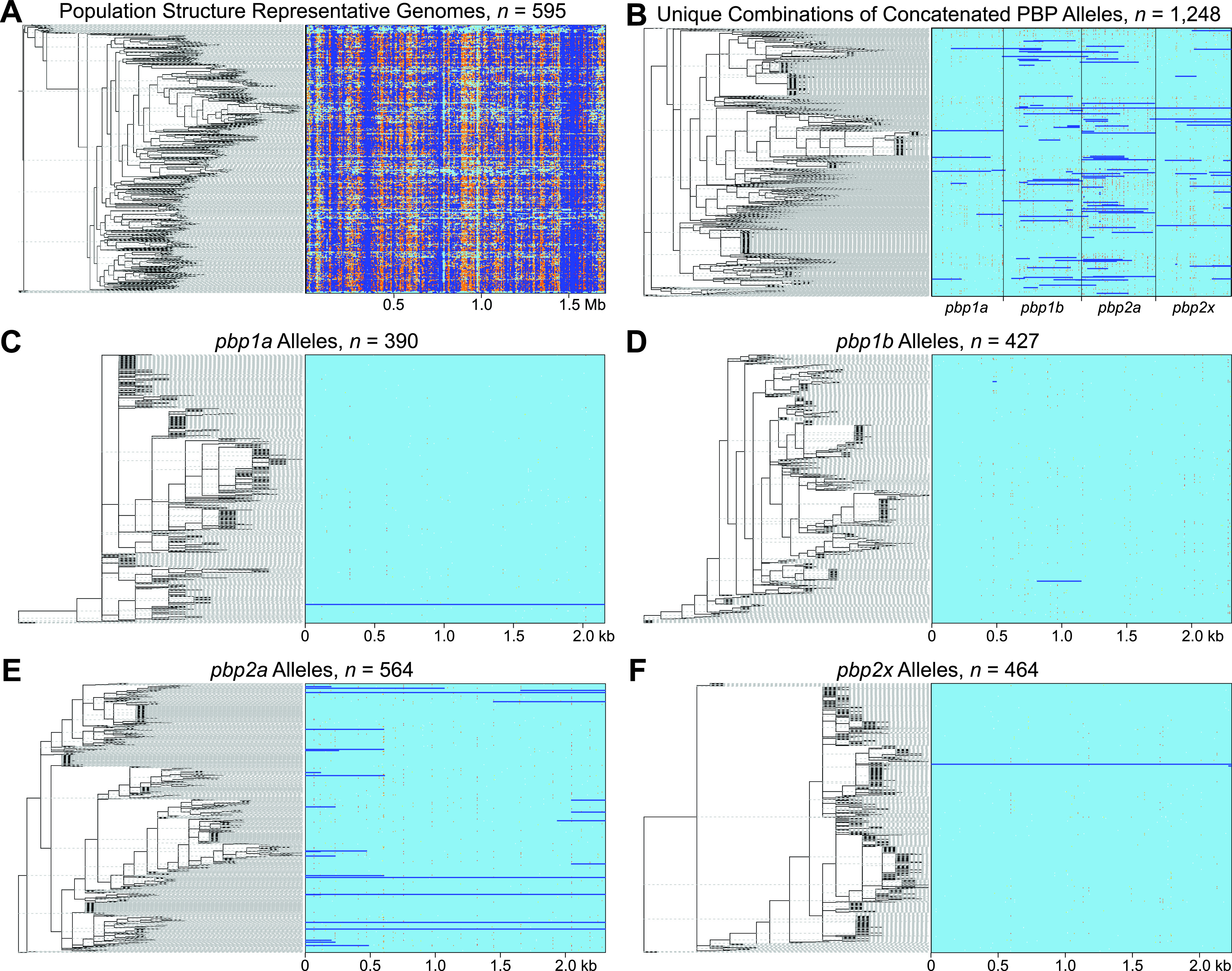
Recombination genome-wide and in the HMM PBPs. (A to F) In each panel, the inferred phylogenies are shown on the left, and the corresponding aligned DNA sequences are shown on the right. In the aligned sequences, recombinant sites are shown in dark blue, invariant sites in light blue, and variant nonhomoplasic sites in white. Variant homoplasic sites are shown from low to high in a yellow-to-red color gradient. Sequence sets analyzed are described at the top of each panel. The single whole-gene recombinant allele identified among the *pbp1a* alleles (panel C) is an added genetic outgroup from SDSE strain NCTC9414. Without this outgroup, meaningful output was not produced, probably because no recombinations were detected in its absence. The single long recombinant segment identified among the *pbp1b* alleles (panel D) is the SDSE-like mosaic genetic outlier allele *pbp1b*-143, and the single whole-gene recombinant identified among the *pbp2x* alleles (panel F) is the SDSE-like genetic outlier allele *pbp2x*-286.

To analyze the relative contribution of recombination and mutation in the evolution of S. pyogenes genes and to place the level of recombination in the HMM PBPs in context, recombination was inferred genome-wide for the population structure representative set of 595 isolates (PSR-595) using ClonalFrameML (47). In the aggregate, 84,094 recombinations were detected along the branches of the inferred phylogeny (Fig. 6A). These recombinations occurred at 22,750 unique start positions, occurring on average every 75 bp across the core genome. The average length of a recombinant segment was 286 bp (range = 2 to 7,956 bp) and had an ANI of 98.9% with the replaced endogenous sequence of the recipient. This high level of identity between the recipient and donor sequences means that the vast majority of the inferred recombination events are likely intraspecific, that is, the sequence donor is another genetic lineage/*emm*-type of S. pyogenes. The genome-wide rate of recombination to mutations (R/Θ) was 0.36, or spontaneous mutations happened about three times as frequently as detected recombination events. However, because the average recombination event introduces multiple SNPs, the relative effect of recombination to mutation (r/m) was 1.69. So, nucleotide changes in the core genome were more likely to arise by homologous recombination than by spontaneous point mutation. In the aggregate, along the branches of the phylogeny the average gene was part of a recombinant segment at a frequency of 66.2 per kb of sequence. The frequency of recombination involving the PBP genes was approximately 10-to-50-fold lower (in descending order) at 6.89 for *pbp1b*, 5.56 for *pbp2a*, 2.31 for *pbp1a*, and 1.33 for *pbp2x*. This low level of recombination in the PBPs relative to the average core gene is consistent with the low level of nsSNPs found and the *dN*/*dS* < 1 site ratios, results suggesting predominantly purifying selection acting on the PBPs. Thus, despite lacking natural competence, recombination was extensive for the genetically diverse S. pyogenes genomes evaluated. Further, recombination involving the PBPs was detected in this subset of the cohort, albeit at a significantly lower frequency than for the average gene.

To assess the relative contributions of recombination and mutation to nucleotide changes and to explore the nature of the donor DNA in HGT events, recombination was analyzed using the PBP allele sets, again using ClonalFrameML. To distinguish between recombination crossover events occurring between the PBP genes, resulting in the reassortment of the PBP alleles into new combinations, and crossovers occurring within a gene, generating mosaic PBP alleles, recombination was analyzed using all unique combinations of the four PBP alleles concatenated end to end (i.e., *pbp1a* + *pbp1b* + *pbp2a* + *pbp2x*) (Fig. 6B) and the allele sets of the individual PBPs separately (Fig. 6C to F), respectively. Substantially more recombinations (ca. 2- to 17-fold) were detected along the branches of the phylogeny for both the population structure representative set of 595 genomes (PRS-595) and the 1,248 unique combinations of the concatenated PBP alleles (pbpcat-1248) than for the phylogenies of the PBP alleles evaluated individually (Table 5). This shows that many of the polymorphisms in the PBPs that were inconsistent with the clonal frame for the phylogeny of the PSR-595 and pbpcat-1248 sequence sets were not inconsistent with the clonal frame for the phylogenies of the respective PBPs individually. This is consistent with most recombination events identified in the PRS-595 and pbpcat-1248 sequence sets being intraspecies and resulting in reassortment of the PBP alleles. This interpretation is consistent with the determined model parameters (Table 5) that show low values for the rate of nucleotide differences in the recombined segments (ν) except for the *pbp1a*-390, *pbp1b*-427 and *pbp2x*-464 allele sets which each include a genetically more distant SDSE sequence. The relative effect of recombination to mutation (r/m) was greater than 1 for all sequence sets analyzed, showing that nucleotide changes were more likely to arise from recombination events than spontaneous mutation events. Because many intraspecies recombination events were not detected when analyzing the alleles of the PBPs individually relative to concatenated PBPs, we believe the model parameters derived from the concatenated sequences more accurately reflect the relative contributions of recombination and mutation to the evolution of the PBPs.

**TABLE 5:** HMM PBP recombination and mutation

Data set	Inferred no. of recombinations					ClonalFrameML model parameters^*a*^					
	1A	1B	2A	2X	Total	R	Θ	R/Θ	δ	ν	r/m
PSR-595	5	14	13	3	35	344,080	967,091	0.356	109.8	0.043	1.69
*pbpcat*-1248	11	35	43	29	118	3,093	1,581	1.957	221.9	0.006	2.49
*pbp1a*-390^*b*^	1	–	–	–	1	1.4	170.7	0.008	3,084.5	0.188	4.69
*pbp1b*-427	–	2	–	–	2	7.2	243.3	0.029	227.8	0.157	1.06
*pbp2a*-564	–	–	27	–	27	1,498	149.5	10.022	193.2	0.003	5.97
*pbp2x*-464	–	–	–	2	2	2.1	199.9	0.010	3,018.7	0.192	6.01

a Model parameters: R, rate of recombination; Θ, rate of mutation; R/Θ, rate of recombination to mutation; δ, mean of the exponential distribution modeling the length of recombined segments; ν, rate of nucleotide differences in the recombined segments; r/m, relative effect of recombination to mutation (R/Θ × δ × ν).

b Values were determined with an added outgroup, *pbp1a* from SDSE strain NCTC9414. Without this outgroup, there were no detected recombination events and ClonalFrameML failed to produce meaningful output.

### Acquisition of foreign HMM PBP2B by horizontal gene transfer.

**FIG 7:**
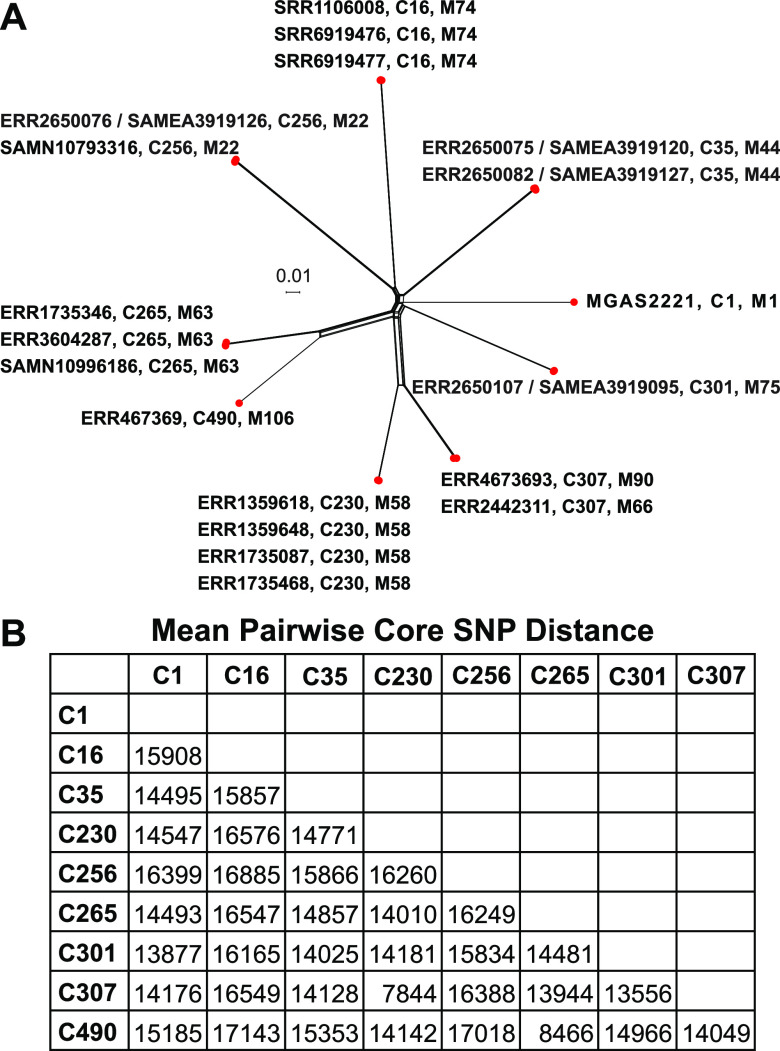
Genetic relationships among S. pyogenes PBP2B recombinant genomes. (A) Genetic relationships among 18 S. pyogenes isolates that have acquired a PBP2B xenologue. Phylogeny was inferred by neighbor-network analysis based on 44,114 core chromosomal SNPs identified relative to Emm1 reference strain MGAS2221. (B) Mean pairwise core SNP genetic distance between the eight different genetic lineages of the PBP2B recombinant isolates. C, cluster designation.

**FIG 8:**
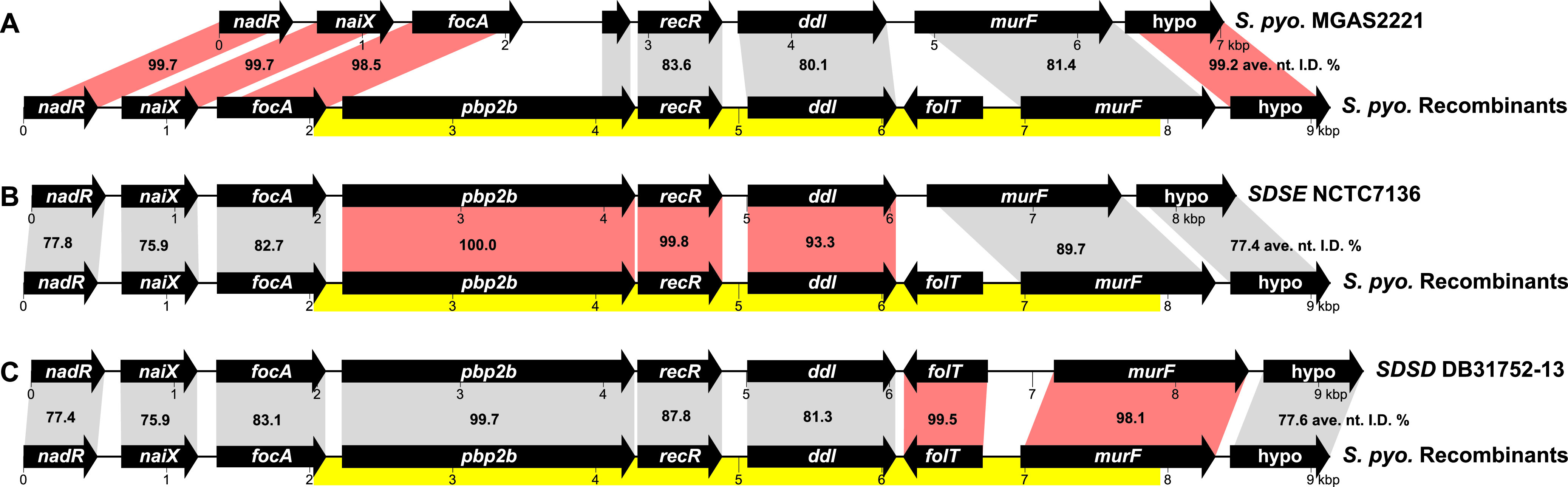
Comparison of streptococcal *pbp2b* regions. Illustrated are comparisons of the S. pyogenes *pbp2b* recombination region gene content with S. pyogenes strain MGAS2221 genome that is representative of wild-type gene content and encodes only a degraded 3′-end fragment of *pbp2b* (A), the SDSE strain NCTC7136 genome (B), and the SDSD strain DB31752-13 genome (C). For each alignment, the average nucleotide identity (ANI) is specified for the genes of the 18 S. pyogenes *pbp2b* recombinant strains with the respective comparison strains. Gene comparisons with the highest identity, suggesting the likely species donor source, are indicated in red. The deduced 5,879-bp recombination region highlighted in yellow is 99.9% identical among the 18 recombinant strains (differing at only 5 SNP sites), consistent with the recombination region sequences sharing a more recent common ancestor than the flanking genes among these closely related streptococci. *S. pyo*., S. pyogenes; SDSE, Streptococcus dysgalactiae subsp. *equisimilis*; SDSD, Streptococcus dysgalactiae subsp. *dysgalactiae*.

Another way in which recombination has been found to contribute to the emergence of β-lactam resistance is through HGT acquisition of exogenous low-affinity PBPs that are not part of the bacteria’s endogenous gene content (8). One important example of this is the emergence of methicillin-resistant Staphylococcus aureus (MRSA), which arose through the HGT acquisition of the exogenous *mecA* gene encoding a class B PBP with low affinity for virtually all β-lactam antibiotics (10). In contrast to S. pyogenes, other streptococci of groups A, B, C, and G (i.e., S. agalactiae, Streptococcus canis, SDSE, *S. dysgalactiae* subsp*. dysgalactiae*, Streptococcus equi subsp*. equi*, and S. equi subsp*. zooepidemicus*) have five endogenous HMM PBPs. Along with orthologues of PBP1A, PBP1B, PBP2A, and PBP2X, these streptococci also encode PBP2B, a second class B PBP not native to S. pyogenes. Given our identification of the interspecies HGT acquisition of SDSE-like *pbp1b* and *pbp2x* sequences (Fig. 2B and D), we analyzed the 26,465 genome assemblies for evidence of acquisition of PBP2B using BLAST. This analysis identified 22 sequence assemblies from 18 unique isolates (4 isolates had sequences deposited in both the SRA and MGDB) with a *pbp2b* xenologue having 100% nucleotide identity with *pbp2b* of SDSE strain NCTC7136. These 18 isolates (Fig. 7) were of 8 different genetic lineages and 9 different *emm*-types: 22, 44, 58, 63, 66, 74, 75, 90, 106, differing lineage to lineage on average by 14,860 core SNPs (range = 7,844 to 17,143). Inspection of the assemblies for these 18 isolates showed the PBP2B gene to be integrated into the S. pyogenes genome in the same chromosomal context as in SDSE and the other pyogenic beta-hemolytic streptococci (Fig. 8). The PBP2B gene is not part of an obvious mobile genetic element, since it is not flanked by sequences associated with plasmids, transposons/insertion sequences, prophages, or integrative conjugative elements. This finding is consistent with recombination of the exogenous PBP2B gene into these 18 isolates of different genetic lineages being mediated by endogenous homologous sequences flanking the site of integration.

The identification of the PBP2B xenologue in multiple divergent S. pyogenes genetic backgrounds could be the result of several independent interspecies HGT events, with each event involving genetically distinct donor and recipient lineages, (i.e., the multiple independent acquisition hypothesis). Alternatively, there could have been a combination of an initial interspecies gene transfer followed by intraspecies HGT-mediated dissemination of the recombined segment to different lineages (i.e., the single dependent acquisition hypothesis). To distinguish between these two hypotheses, we examined the crossover boundaries of the mosaic sequences and the extent of genetic diversity present in the recombined segments of the 18 isolates. This comparison identified a 5,879-bp region that was virtually identical in nucleotide sequence (>99.9% ANI, differing at only 5 SNP sites) among the 18 recombinants (Fig. 8). The high level of sequence identity (1 SNP on average every 1,176 bp) is ∼10-fold greater than the ANI for the S. pyogenes core genome from one lineage/*emm* type to another (∼1 SNP every 120 bp) and is greater than that of the flanking sequences. No additional nonnative sequence flanking this region of high identity was found, consistent with the 18 isolates having the same or very similar HGT/recombination boundaries. It is unlikely that multiple independent interspecies HGT events would result in the same recombination crossover boundaries and involve virtually identical donor recombinant segments among the 18 isolates. Therefore, the sequence data are most parsimonious with the acquisition of the PBP2B xenologue occurring through an initial interspecies HGT event into a single isolate followed by dissemination to isolates of other genetic lineages through multiple subsequent intraspecies HGT events. This subsequent intraspecies dissemination was likely evolutionarily fairly recent because of the lack of sequence divergence in the recombinant segment in isolates of different genetic lineages.

The 5.9 kbp recombined segment encodes five proteins: PG synthesis transpeptidase/penicillin binding protein PBP2B, DNA repair and recombination protein RecR, PG precursor synthesis d-alanyl-d-alanine ligase Ddl, folate transport protein FolT, and PG precursor synthesis UDP-MurNAc-tripeptide—d-alanyl-d-alanine ligase MurF (Fig. 8). The donated DNA appears to be an evolutionary mosaic stemming from more than a single S. dysgalactiae donor subspecies. The 5′ end of the donated sequence encoding *pbp2b-recR-ddl* has high identity with SDSE strain NCTC7136 sequence (Fig. 8B), whereas the 3′ end encoding *folT-murF* has high identity (>98%) with S. dysgalactiae subsp*. dysgalactiae* strain DB31752-13 sequence (Fig. 8C). In comparing the 5.9-kb donated segment with the NBCI sequence database, only SDSD strain DB31752-13 had a full-length match containing all five genes. This is a little perplexing in that *folT* is not part of the endogenous core of S. pyogenes, SDSE, or SDSD genomes, suggesting that the presence of *folT* in the genome of SDSD strain DB31752-13 is also likely due to an exogenous HGT acquisition. In conclusion, although S. dysgalactiae is the likely source of the recombinant segment, we did not identify a single donor with high sequence identity across the entire 5.9-kb sequence. The consequence of this PBP2B acquisition on β-lactam susceptibility is not known and requires further investigation. However, the presence of an exogenous SDSE-like *pbp2B* xenologue, along with the acquisition of an endogenous SDSE-like *pbp2x* orthologue, clearly demonstrates that if impediments to HGT exist in S. pyogenes, they are not severe enough to prevent interspecies acquisition of HMM PBPs homologues and subsequent rapid intraspecies dissemination.

### Unexpected discovery of a chimeric PBP2X protein that confers decreased susceptibility to multiple β-lactam antibiotics.

**FIG 9:**
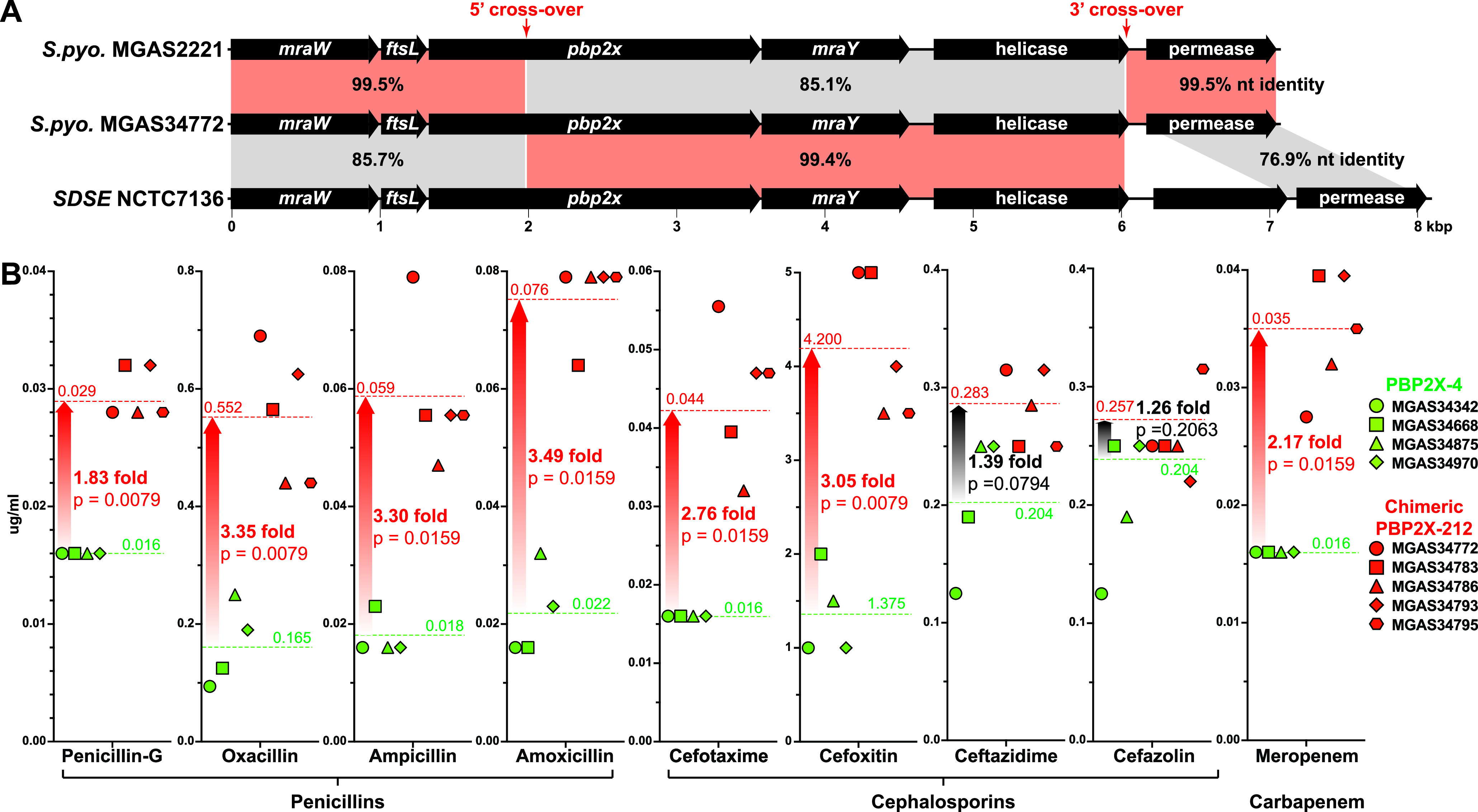
Comparison of recombinant *emm81* strains encoding chimeric PBP2X-212 with reference strains. (A) Comparison of the gene content and nucleotide identity of the *pbp2x* regions of S. pyogenes reference *emm1* strain MGAS2221, recombinant *emm81* strain MGAS34772 encoding chimeric PBP2X, and SDSE strain NCTC7136. Aligned sequences are highlighted, with regions sharing highest nucleotide identity indicated in red. (B) Compared are MICs determined for four strains encoding PBP2X-4 the most prevalent variant among *emm81* isolates shown in green, and five strains encoding chimeric variant PBP2X-212 shown in red, using a panel of nine β-lactam antibiotics. Graphed are average MICs determined from Etests done in duplicate. Mean MIC values for the wild-type PBP2X-4 group and the chimeric PBP2X-212 group are provided and indicated by dotted lines. The statistical significance of differences between the groups was evaluated using the nonparametric two-tailed Mann-Whitney test. The fold change is expressed as the ratio of the greater group mean to the lesser group mean. *S. pyo.*, S. pyogenes; SDSE, Streptococcus dysgalactiae subsp. *equisimilis*.

As the present study was being readied for submission, while sequencing isolates collected in French Brittany (48), we unexpectedly discovered a group of five *emm81* strains that had a highly divergent *pbp2x* allele not present in the cohort of 26,465 WGSs. These five strains are clonally related, differing from each other on average by only 28 core SNPs. In contrast, the five strains differ on average by 3,177 core SNPs from isolates of the most prevalent *emm81* genetic lineage of the cohort (i.e., genetic cluster 21). Analysis of genome assemblies for these five strains identified an 100% ANI 4-kbp SDSE-like recombinant segment that results in a chimeric *pbp2x*, designated allele *pbp2x-*465 (Fig. 9A). The product of *pbp2x*-465, designated PBP2X-212, is SDSE-like over amino acids 200 to 751 (i.e., 551 amino acids) and differs from PBP2X-1, the most prevalent PBP2X variant, by 55 amino acid substitutions (see Fig. S2). The majority of these substitutions (30 of 55) are within the TP domain, but none are in the three conserved catalytic motifs. In contrast, the transpeptidase domain of chimeric PBP2X-212 differs from horizontally acquired SDSE strain NCTC7136-like variant PBP2X-112 by three substitutions: A_511_V, M_593_T, and G_600_D (Fig. 4D; see also Fig. S2). Substitutions PBP2X M_593_T and G_600_D have both been proven to result in reduced penicillin susceptibility (Table 4). To assess the potential consequences of the recombination event on β-lactam susceptibility, the five *emm81* chimeric PBP2X-212 strains were compared to four genetically related *emm81* cluster C-21 strains encoding PBP2X-4, the most common variant found among *emm81* isolates of the cohort. Importantly, with a single exception, these four *emm81* comparator strains have the same PBP 1A, 1B, and 2A variants as the five recombinant chimeric isolates (see Table S1). Therefore, differences in β-lactam susceptibility between the *emm81* chimeric PBP2X-212 group and the PBP2X-4 group, cannot be attributed to sequence differences in PBPs other than PBP2X. Relative to the four genetically related comparators, all five chimeric PBP2X-212 isolates have statistically significantly 1.83- to 3.49-fold-increased average MICs for seven of nine β-lactam antibiotics evaluated (Fig. 9B). Experimental proof that chimeric PBP2X-212 is sufficient to cause these MIC increases will require the construction of an isogenic mutant strain.

10.1128/mbio.03618-21.2 FIG S2 Aligned PBP2X sequences. Illustrated are sequences of the most prevalent S. pyogenes PBP2X variant (PBP2X-1), the chimeric variant (PBP2X-212), and PBP2X from SDSE strain NCTC7136 aligned with ClustalW. Regions of the alignment sharing identity are highlighted in red. Amino acids of the consensus sequence (below) are highlighted by functional domains as indicated in the inset lower right. The three transpeptidase key conserved catalytic motifs (SXXK, SXN, and KSGT) are shown in red. Download FIG S2, PDF file, 1.3 MB. Copyright © 2022 Beres et al. 2022 Beres et al. https://creativecommons.org/licenses/by/4.0/ This content is distributed under the terms of the Creative Commons Attribution 4.0 International license.

## DISCUSSION

Here, we defined the genetic diversity present in the most comprehensive collection of S. pyogenes whole-genome sequences studied to date. The population genomic data set facilitated development of an integrative reverse genotype-to-phenotype investigative strategy that was exploited to yield new knowledge about genetic polymorphisms that decrease antimicrobial agent susceptibility, a global public health problem of immense magnitude. Given the technical ease and low cost of large-scale microbial whole-genome sequencing and access to large, publicly available genome databases, the strategy we used is broadly applicable to other pathogens.

Despite the cohort encompassing much of the currently available WGS data for the species, 26,465 samples constitute only a very small fraction (0.004%) of the ∼700 million infections caused annually by this human pathogen. This fact, along with the finding that 30 to 40% of the PBP alleles were unique to a single isolate, argues that only a very minor portion of the species’ genetic diversity is represented in this relatively large genomic cohort. Moreover, because most of the isolates are of unknown provenance and not available for confirmatory genotypic or phenotypic testing, only limited epidemiologic and pathogenic correlations can be made. In addition, the cohort is biased because it is composed largely of sequences of isolates collected in studies of invasive infections predominantly in economically developed countries. The great majority of GAS infections treated with β-lactams are throat and noninvasive skin infections, the highest burden of which is in less economically developed countries (3). Inasmuch as Sec-dependent protein translocation and sortase mediated cell wall anchoring are linked with nascent PG synthesis, there is reason to suspect and some evidence to support that mutations in PBPs that decrease β-lactam susceptibility in S. pyogenes, may perturb the cell surface localization of some key virulence molecules (e.g., M protein, pilus, and hyaluronate capsule) (49, 50). Thus, if antibiotic selection for reduced penicillin susceptibility occurs primarily in noninvasive infections and contributes to a reduced capacity to cause invasive infections, then invasive infection isolates may not adequately represent the span of PBP diversity and mutations associated with reduced β-lactam susceptibility. Cognizance of these limitations is relevant to interpreting our findings and highlights aspects in need of further investigation.

One goal of the present investigation was to identify amino acid substitutions in HMM PBPs that may confer decreased susceptibility to β-lactam antibiotics. To that end, we identified 92 PBP1A variants not previously described, 81 new PBP1B variants, 141 new PBP2A variants, and 112 new PBP2X variants. Among the four PBPs, variation was found at 20 to 27% of amino acid positions, a substantial portion of which were located in the penicillin-binding transpeptidase domain and have potential for altering β-lactam interaction and decreasing susceptibility. Four key substituted sites located in one of the three transpeptidation conserved catalytic motifs of PBP2X (Fig. 5) are of special interest: (i) T_341_A and M_342_I adjacent to the catalytic serine within the S_340_XXK motif, (ii) S_400_R in the S_399_XN motif, and (iii) a second example of the T_553_K substitution in the conserved K_550_SGT motif. This second example of the T_553_K substitution was found in an *emm6* strain (sample ERR1516025) that differs in PBP2X allele/variant from the two *emm43* isolates identified by Vannice et al. (28). WGS data for this *emm6* strain were deposited in the SRA in 2016 and therefore its isolation predates the 2017-2018 identification of the isolates documented by Vannice et al. Two substitutions, T_459_A and K_461_N, were identified in the S_458_XXK motif of PBP2A. No substitutions were identified in the catalytic motifs of PBP1A or PBP1B. Some of the substituted sites identified in the PBP2X transpeptidation catalytic motifs also have been identified at high frequency among penicillin-resistant isolates of S. pneumoniae (8, 51). In contrast, substitutions at V_402_ adjacent to the S_399_XN motif and at Q_555_ adjacent to the K_550_SGT motif are notably absent among the 211 PBP2X variants in S. pyogenes. Mutations at one or both of these corresponding sites are prevalent among resistant S. pneumoniae and S. agalactiae isolates (18, 21, 52–57) and were identified in resistant S. dysgalactiae subsp. *equisimilis* and S. gordonii isolates (23, 58). Determination of the three-dimensional structures of the S. pyogenes HMM PBPs and their variants will be crucial to provide enhanced understanding of how variant residues alter β-lactam susceptibility in S. pyogenes relative to other streptococci and for interpreting structure-function relationships of other substitutions identified with signatures of positive selection.

Historically, investigations identifying genetic changes that alter antimicrobial resistance have started with an observed phenotype (decreased antibiotic susceptibility) and moved to genotype usually by targeted genetic analysis or WGS. That is, resistant isolates are identified first via standard susceptibility testing and then the genes or genomes of the isolates are sequenced and compared with those of a wild-type susceptible reference strain to identify resistance-associated mutations. Isogenic mutant strains are then constructed to directly test the capacity of candidate polymorphisms to alter antibiotic susceptibility. In some investigations this approach has been taken a step further in that sites nonrandomly associated with resistant strains have also been shown to have signatures of positive selection such as a *dN*/*dS* ratio greater than 1 in the case of S. pneumoniae (37) or having arisen through convergent evolution in the case of Mycobacterium tuberculosis (38). Here, we have leveraged these previous studies demonstrating that resistance sites correlate with positively selected sites to take the converse approach of identifying sites under positive selection to infer sites involved in resistance. We have used this reverse-genotype-to-inferred-phenotype approach in an attempt to overcome the lack of antibiotic susceptibility information available for the rich genetic information available in the deposited genome sequences. In the case of PBPs, the genotype to phenotype inference is based on the fact that β-lactams exert evolutionary pressure on the PBP transpeptidase domain, selecting for substitutions that reduce affinity for the antibiotics (37, 59). Our analysis showed that PBPs are evolving under strong purifying selection. Although many codon sites of the PBPs were identified as evolving under significant negative selection, importantly, only 24 sites in total among all four of the PBPs had signals of evolving under significant positive selection. Since isolates with these newly identified candidate substitutions are not part of our strain collection, we could not evaluate their predicted association with reduced susceptibility. However, for the 11 PBP2X sites previously associated with reduced β-lactam susceptibility *in vitro* (28, 30, 31), there was a strong correlation with positively selected codon sites. Of these 11 sites, 8 had a positive *dN*/*dS* ratio (Table 4), which is consistent with a positive *dN*/*dS* ratio being a good predictor of association between amino acid substitutions in the PBP transpeptidase domain and reduced β-lactam susceptibility (37). Moreover, seven of eight isogenic mutant strains generated for PBP2X substitutions at six sites with signatures of positive selection had increased penicillin MICs. These findings raise the strong possibility that the 52 other untested sites with signatures of positive selection in the transpeptidase domain of the four HMM PBPs will also mediate decreased susceptibility to β-lactam antibiotics. Investigation of this will require construction and analysis of additional isogenic mutant strains, and these studies are under way.

One posited explanation for the lack of penicillin resistance emergence in S. pyogenes is a low level of effective recombination, usually attributed to a lack of natural competence (6, 29). However, our study shows that recombination has contributed to shaping genetic diversity in the species, a finding consistent with multiple studies using MLST data which have found S. pyogenes to have a level of recombination comparable to that of S. pneumoniae and greater than that of Neisseria meningitidis, two naturally competent species (60–63). In addition, assessment of core genome recombination for 36 S. pyogenes genetic lineages found an effective recombination/mutation (r/m) ratio of 4.95 (64). As with these prior investigations, we identified extensive recombination throughout the core genome among the 595 isolates representing the diverse population structure of S. pyogenes, including recombination involving each of the PBPs. The PBPs as a group have an r/m ratio of 2.49, meaning that SNPs were more likely to arise from recombination than mutation. The average recombinant donor segment shared high sequence identity with the replaced recipient segment, indicating that most recombination events were intraspecific or involved genetically closely related species. The length and high average nucleotide identity of the recombinant donor sequences are consistent with generalized phage transduction being the primary mechanism of HGT/recombination. Consistent with this, genome sequencing has found evidence supporting HGT/recombination between and a commonality of phages among group A, C, and G streptococci (24, 65–69). This overlapping phage host range raises the possibility that if a β-lactam-resistant PBPs were to evolve in any of these species (S. dysgalactiae, S. equi, or S. pyogenes), then generalized transduction could transfer it to all of them. This is concerning given that β-lactam resistance has been reported in SDSE (23). This concern is magnified by our unexpected finding of five *emm81* strains with a chimeric PBP2X resulting from HGT acquisition of SDSE-like sequences, that have approximately 2- to 4-fold-increased MICs for multiple β-lactam antibiotics. Of note, two of these *emm81* isolates came from cases of recurrent infection with treatment failure.

Our study identified the first examples of intraspecies recombination involving S. pyogenes *pbp1b* and *pbp2x* genes (Fig. 3). This finding establishes that S. pyogenes mosaic PBPs can evolve through intraspecies HGT events and that the recombinants are of sufficient fitness to transmit and cause invasive infection. Moreover, for the five *emm81* strains with chimeric PBP2X, it shows that these intraspecies HGT events can result in significantly decreased β-lactam susceptibility. Interspecies recombination generating mosaic PBPs is the primary process by which β-lactam resistance in S. pneumoniae evolved (15, 17). Of additional concern is the identification of 18 isolates in eight different genetic lineages that have acquired an exogenous *pbp2b* gene by HGT. This *pbp2b* gene is identical to one in SDSE. Although the fitness effect of having this additional class B PBP gene is unknown, its presence in multiple diverse genetic lineages suggests that it may confer a survival advantage.

It has recently been established that PBP2B transpeptidase activity functions in conjunction with RodA transglycosylase activity as part of the elongasome and that PBP2X transpeptidation functions with FtsW transglycosylation as part of the divisome to catalyze lateral wall and septal PG synthesis, respectively (70, 71). Because S. pyogenes genomes lack *rodA*, *mreC*, and *mreD*, all components of the elongasome, the extent to which PBP2B in the absence of these other components may function in PG synthesis is unclear. However, we note that horizontal acquisition of an exogenous low affinity class B PBP, namely, MecA, is how methicillin resistance emerged in S. aureus (10). Although speculative, it is possible that acquisition of PBP2B provides some degree of transpeptidase activity redundancy and lowers the purifying functional constraints acting on PBP2X, the lone class B PBP in S. pyogenes.

In summary, our population genomic study of the diversity and evolution of the HMM PBPs reveals strong purifying selection and biased recombination as evolutionary constraints likely impeding the emergence of β-lactam resistance in S. pyogenes. Despite these impediments, the finding of intraspecies recombinant PBP1B and PBP2X sequences and acquisition of exogenous PBP2B suggests that emergence of β-lactam resistance in S. pyogenes may be a single HGT event away. The strong correlation between PBP2X codons with signals of positive selection and substitutions that in isogenic mutants confer increased β-lactam MICs shows the integrated reverse population genomic analysis of evolutionary selection strategy employed here to successfully identify PBP2X variants with decreased antibiotic susceptibility. Our identification of scores of additional experimentally untested PBP transpeptidase domain substitutions with signatures of positive selection strongly supports the existence of unrecognized molecular variants that decrease β-lactam susceptibility. The effect of these substitutions in PBP2X and the other HMM PBPs on β-lactam susceptibility is unknown but warrants study.

## MATERIALS AND METHODS

### Genomic sequencing data.

Genomic sequencing read sets were obtained from the National Center for Biotechnology Information Sequence Read Archive (NCBI SRA; https://www.ncbi.nlm.nih.gov/sra) by searching with the terms S. pyogenes, whole-genome sequencing (WGS), and Illumina platform. This identified 49 BioProjects (all but PRJNA559889 having WGS runs for 10 or more isolates) containing 25,045 runs of Illumina WGS single or paired-end reads. The NCBI Microbial Genome Database (MGDB; https://www.ncbi.nlm.nih.gov/genome/browse#!/prokaryotes/) was searched for genome assemblies (partial and complete) with the term S. pyogenes. This identified 2,123 WGS assemblies (210 complete closed and 1,913 partial contigs/scaffolds). Sequences were downloaded using SRAToolkit fasterqdump (https://github.com/ncbi/sra-tools). There is some redundancy in the WGS data cohort, stemming from isolates for which sequence data were deposited in both the SRA and MGDB. No attempt was made to cross reference the databases and remove redundant samples which, if entirely overlapping, would maximally represent 15.6% of the cohort. Retention of this redundant WGS data provided an important internal control for subsequent genetic characterizations (i.e., *emm* type, MLST, core SNPs, lineage, PBP alleles, etc.) as the same determinations should be made for the duplicated sequence samples. Variation in the PBPs, largely focused on the transpeptidase domain of PBP2X, has been studied previously for some of these samples (5, 28–31).

### Whole-genome sequence assembly.

Unassembled WGS read sets obtained from the NCBI SRA (*n *=* *25,045) were filtered to remove low-quality, adapter, and library generation artifact sequences using Trimmomatic (72) and were base call error corrected using Musket (73). After this preprocessing, the WGS read sets were assembled *de novo* with SPAdes (74). The genomic assemblies thus generated were combined with the genome assemblies obtained from the NCBI MGDB (*n *=* *2,123) and statistics (i.e., number of contigs, total assembled base pairs, *N*_50_, G+C%) were gathered for the combined 27,168 genomic assemblies using SeqKit stats function (75) (see Table S1). WGS read sets that failed to assemble (*n *=* *6), that were under sequenced (*n *=* *147, assemblies < 1.5 Mbp), that were likely contaminated (*n *=* *490, assemblies > 2.2 Mbp), or that were not S. pyogenes (*n *=* *60) were excluded.

### Epidemiological molecular markers.

As an initial assessment of the composition of the cohort the traditional epidemiological markers, *emm* type and multilocus sequence type (MLST) was determined for all samples. Emm/M type determination from short reads is problematic since some lineages of S. pyogenes encode in addition to Emm/M protein, an M-related protein (Mrp) and an Emm-like protein (Enn), whose genes can share sequence similarity confounding read mapping and assembly. To enhance determination of these epidemiological markers, both read mapping-based, with unassembled reads and ShortRea*dS*equenceTyper2 (76), and BLAST-based, with assembled reads and EmmTyper (https://github.com/MDU-PHL/emmtyper) and MLSTcheck (https://github.com/sanger-pathogens/mlst_check), approaches were used. Assignments of *emm* type were made relative to the CDC *emm* database (https://www2.cdc.gov/vaccines/biotech/strepblast.asp) and MLST relative to the PubMLST database (https://pubmlst.org/organisms/streptococcus-pyogenes). Samples for which an *emm* type and MLST could not be determined were suspected of being contaminated or not S. pyogenes and were purged from the cohort.

### SNP discovery and phylogenetic reconstruction.

SNPs were identified by comparison of each genomic assembly to reference genomes, M89 strain MGAS23530 and M1 strain MGAS2221, using the MUMmer dnadiff function (77). SNPs were concatenated and converted to an aligned multifasta sequence file using Prephix and Phrecon (https://github.com/codinghedgehog) and were annotated using snpEFF (78). Phylogeny for the cohort was inferred by neighbor-joining using RapidNJ (79), and trees were generated using Dendroscope (80).

### Population structure determination.

Pseudo core genomes were generated for all 26,465 samples of the curated cohort using SNPswapper (https://github.com/codinghedgehog) with SNPs identified relative to the genome of reference M89 strain MGAS23530 substituted back into the reference genome. Strain MGAS23530 lacks phages and ICEs, and its sequence is therefore essentially limited to the core genome. This process results in a pseudo core genome alignment preserving the core genome genetic differences between the sequences and excluding potentially erroneous SNPs identified relative to quasirepetitive mobile genetic element sequences. Distinct genetic lineages within the population were assessed among the aligned pseudo core genomes using PopPUNK (81) by density-based spatial clustering. To facilitate some analyses that were computationally problematic/intractable when applied to the entire cohort of 26,465 WGS read sets, a reduced minimal set of 595 samples broadly representative of the population structure was generated based on the following selection criteria: (i) include samples of each different genetic cluster identified; (ii) if a given cluster included samples of more than one *emm* type or previously determined genetic lineage (for example, preepidemic and epidemic *emm*1 lineages), then additional samples with these characteristics were included; and (iii) preference was given to samples with complete closed WGS assemblies or, if incomplete, a higher *N*_50_ and a lower number of contigs. Pairwise genetic distances in terms of SNP differences between clusters were determined using MEGA (82).

### HMM PBP alleles and variants identification.

PBP gene sequences were identified in the WGS assemblies by BLASTN (83) search using the HMM PBPs of M1 strain MGAS2221 as queries. Search hits were extracted from the WGS assemblies using Bedtools getfasta (84), and sequences encoding in-frame full-length translated products were retained and used to generate for each PBP a nonredundant set of unique alleles (see Table S2). Allele sequences were aligned by the codon aware back translation method using TranslatorX (85). Phylogeny was inferred for the aligned PBP alleles by the method of maximum likelihood using PhyML (86). The PBP unique alleles were translated to their amino acid products from which nonredundant unique protein variant sets were derived. The PBP unique variant amino acid sequences were aligned using ClustalO (87), and phylogeny was inferred for the aligned proteins by maximum likelihood using PhyML. Pairwise genetic distances between alleles and variants were determined using MEGA.

### Recombination, mutation, and selection inference.

ClonalFrameML (47) was used to infer recombined sequence blocks, rates of recombination and mutation for (i) the population structure representative set of 595 aligned core genomes (PSR-595), (ii) the individual HMM PBPs allele sets, and (iii) the 1,248 unique combinations of concatenated PBP alleles (PBPcat-1248), in the order *pbp1a* + *pbp1b* + *pbp2a* + *pbp2x*, present in the cohort. Homoplasic codon sites were identified among PBPcat-1248 aligned sequences using SNPPar (39). PBP allele sets were tested for recombination using GARD (88), and codon sites under pervasive or episodic selection were inferred from the PBP aligned allele sets using FUBAR (42), MEME (41), and SLAC (40) implemented in HyPhy2.5 (89).

### PBP2X isogenic mutant strain construction and characterization.

Isogenic strains with amino acid substitutions in PBP2X were constructed as previously described (31). The PBP2X substitutions were constructed in *emm89* parental strain MGAS27213-L_601_P, which produces the most prevalent wild-type PBP2X-1 variant. Primers used to construct the isogenic strains are provided in Table S8. Isogenic strains were whole genome sequenced to confirm the PBP2X substitution construction and a lack of spurious spontaneous mutations. MICs for β-lactam antibiotics were determined using the Etest gradient method (bioMérieux, Marcy-l’Étoile, France).

10.1128/mbio.03618-21.10 TABLE S8 PBP2X substitution isogenic construct primers. Download Table S8, DOCX file, 0.02 MB. Copyright © 2022 Beres et al. 2022 Beres et al. https://creativecommons.org/licenses/by/4.0/ This content is distributed under the terms of the Creative Commons Attribution 4.0 International license.

### Data availability.

Data for the nine S. pyogenes *emm81* strains whole genome sequenced as part of this investigation have been deposited in the NCBI SRA under BioProject PRJNA785543. All other data are included in the tables, figures, and supplemental material.
