# Supplementary material for: Integrative Reverse Genetic Analysis Identifies Polymorphisms Contributing to Decreased Antimicrobial Agent Susceptibility in Streptococcus pyogenes
Source: mBio. 2022 Jan 18;13(1):e03618-21. doi: 10.1128/mbio.03618-21 (PMC8764543; doi:10.1128/mbio.03618-21)
Supplement: FIG S1 [file mbio.03618-21-sf001.pdf]

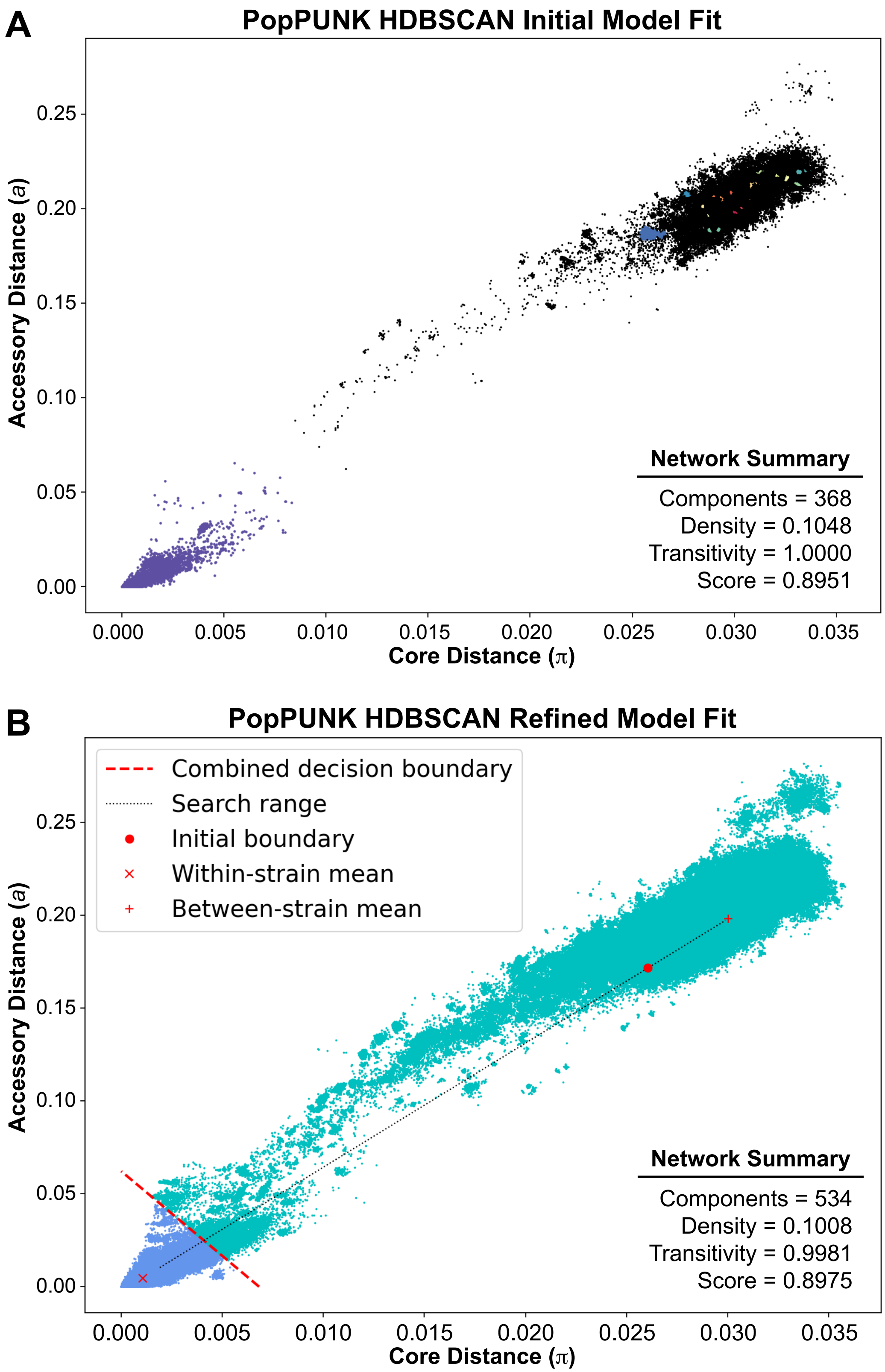


**FIG S1** Determination of *S. pyogenes* genetic lineages. Initial (A) and refined (B) model fits for hierarchical density-based spatial clustering of 26,465 *S. pyogenes* pseudo core genomes. Pseudo core genomes were generated based on 415,239 core SNPs identified relative to the genome of reference M89 strain MGAS23530. Population partitions were determined using PopPUNK v 2.3.0.
