# Supplementary material for: Integrative Reverse Genetic Analysis Identifies Polymorphisms Contributing to Decreased Antimicrobial Agent Susceptibility in Streptococcus pyogenes
Source: mBio. 2022 Jan 18;13(1):e03618-21. doi: 10.1128/mbio.03618-21 (PMC8764543; doi:10.1128/mbio.03618-21)
Supplement: TABLE S3 [file mbio.03618-21-st003.docx]

**TABLE S3** HMM PBP gene statistics

| **PBP** | **Sequences** | **Seqs % 26,465** | **Min Length** | **Max Length** | **Mode Length** | **Mode Count** | **Mode % Seqs** | **Alleles** |
| --- | --- | --- | --- | --- | --- | --- | --- | --- |
| 1A | 25,721 | 97.19 | 2145 | 2193 | 2166 | 25,430 | 98.87 | 389 |
| 1B | 25,410 | 96.01 | 2301 | 2304 | 2301 | 25,406 | 99.98 | 427 |
| 2A | 25,549 | 96.54 | 2337 | 2337 | 2337 | 25,549 | 100.00 | 564 |
| 2X | 25,271 | 95.49 | 2112 | 2268 | 2256 | 25,237 | 99.87 | 464 |
