# Supplementary material for: Integrative Reverse Genetic Analysis Identifies Polymorphisms Contributing to Decreased Antimicrobial Agent Susceptibility in Streptococcus pyogenes
Source: mBio. 2022 Jan 18;13(1):e03618-21. doi: 10.1128/mbio.03618-21 (PMC8764543; doi:10.1128/mbio.03618-21)
Supplement: TABLE S4 [file mbio.03618-21-st004.docx]

**TABLE S4** HMM PBP gene nucleotide variation statistics

| **Allele set** | **Length (nts)** | **Mono-Allelic** | **Bi-Allelic** | **Tri-Allelic** | **Quad-Allelic** | **Invariant %** | **Variant** | **Variant %** |
| --- | --- | --- | --- | --- | --- | --- | --- | --- |
| *pbp1a*, *n* = 389 | 2166 | 1833 | 321 | 10 | 2 | 84.63 | 333 | 15.37 |
| *pbp1b*, *n* = 426 | 2301 | 1947 | 331 | 22 | 1 | 84.62 | 354 | 15.38 |
| *pbp1b*, *n* = 427 *^a^* | 2301 | 1899 | 373 | 28 | 1 | 82.53 | 402 | 17.47 |
| *pbp2a*, *n* = 564 | 2337 | 1936 | 383 | 17 | 1 | 82.84 | 401 | 17.16 |
| *pbp2x*, *n* = 463 | 2256 | 1925 | 318 | 23 | 2 | 85.33 | 343 | 15.20 |
| *pbp2x*, *n* = 464 *^a^* | 2256 | 1606 | 586 | 71 | 5 | 71.19 | 662 | 29.34 |

*^a^* Allele sets include genetic outlier alleles (*pbp1b*-143 and *pbp2x*-286) with divergent SDSE-like HGT/recombinant sequences.
