# Supplementary material for: Integrative Reverse Genetic Analysis Identifies Polymorphisms Contributing to Decreased Antimicrobial Agent Susceptibility in Streptococcus pyogenes
Source: mBio. 2022 Jan 18;13(1):e03618-21. doi: 10.1128/mbio.03618-21 (PMC8764543; doi:10.1128/mbio.03618-21)
Supplement: TABLE S5 [file mbio.03618-21-st005.docx]

**TABLE S5** HMM PBP gene SNP distribution with respect to the average core gene

|  | **SNPs** *^a^* | | | | | **sSNPs** *^a^* | | | | | **nsSNPs** *^a^* | | | | |
| --- | --- | --- | --- | --- | --- | --- | --- | --- | --- | --- | --- | --- | --- | --- | --- |
| **PBP** | **Obs** | **Exp** | **Obs/Exp %** | **χ2** | **p** | **Obs** | **Exp** | **Obs/Exp %** | **χ2** | **p** | **Obs** | **Exp** | **Obs/Exp %** | **χ2** | **p** |
| *pbp1a* | 357 | 553 | 64.5 | 41.84 | 7.37E-17 | 185 | 237 | 78.1 | 6.17 | 0.012 | 172 | 316 | 54.4 | 41.95 | 5.20E-16 |
| *pbp1b* | 390 | 589 | 66.2 | 39.82 | 2.87E-16 | 214 | 252 | 84.9 | 2.94 | 0.086 | 175 | 336 | 52.1 | 50.16 | 1.49E-18 |
| *pbp2a* | 435 | 598 | 72.7 | 25.49 | 2.45E-11 | 216 | 255 | 84.7 | 3.07 | 0.080 | 218 | 341 | 63.9 | 26.66 | 2.62E-11 |
| *pbp2x* | 369 | 578 | 63.8 | 45.45 | 4.20E-18 | 177 | 247 | 71.2 | 11.24 | 0.001 | 191 | 329 | 58.1 | 36.14 | 2.65E-14 |

*^a^* Abbreviations: Obs = observed, Exp = expected.
