# Supplementary material for: Integrative Reverse Genetic Analysis Identifies Polymorphisms Contributing to Decreased Antimicrobial Agent Susceptibility in Streptococcus pyogenes
Source: mBio. 2022 Jan 18;13(1):e03618-21. doi: 10.1128/mbio.03618-21 (PMC8764543; doi:10.1128/mbio.03618-21)
Supplement: TABLE S6 [file mbio.03618-21-st006.docx]

**TABLE S6** HMM PBP transglycosylase domain SNP distribution *^a^*

|  | **SNPs***^b^* | | | | | **sSNPs***^b^* | | | | | **nsSNPs***^b^* | | | | |
| --- | --- | --- | --- | --- | --- | --- | --- | --- | --- | --- | --- | --- | --- | --- | --- |
| **PBP** | **Obs** | **Exp** | **Obs/Exp %** | **χ2** | **p** | **Obs** | **Exp** | **Obs/Exp %** | **χ2** | **p** | **Obs** | **Exp** | **Obs/Exp %** | **χ2** | **p** |
| *pbp1a* | 75 | 89 | 84.2 | 1.55 | 0.247 | 46 | 46 | 100.0 | 0.02 | 0.904 | 29 | 43 | 67.4 | 2.97 | 0.085 |
| *pbp1b* | 87 | 98 | 88.8 | 0.71 | 0.400 | 56 | 54 | 103.7 | 0.01 | 0.912 | 31 | 44 | 70.5 | 2.44 | 0.118 |
| *pbp2a* | 86 | 96 | 89.6 | 0.56 | 0.453 | 51 | 48 | 106.3 | 0.05 | 0.819 | 35 | 48 | 72.9 | 2.14 | 0.143 |
| *pbp2x* | 73 | 85 | 85.9 | 0.98 | 0.323 | 44 | 41 | 107.3 | 0.06 | 0.803 | 34 | 44 | 77.3 | 1.31 | 0.253 |

*^a^* PBP2X lacks a transglycosylase domain. The data provided is for the PBP2X dimerization domain.

*^b^*Abbreviations: Obs = observed, Exp = expected.
