# Supplementary material for: Integrative Reverse Genetic Analysis Identifies Polymorphisms Contributing to Decreased Antimicrobial Agent Susceptibility in Streptococcus pyogenes
Source: mBio. 2022 Jan 18;13(1):e03618-21. doi: 10.1128/mbio.03618-21 (PMC8764543; doi:10.1128/mbio.03618-21)
Supplement: TABLE S7 [file mbio.03618-21-st007.docx]

**TABLE S7** HMM PBP transpeptidase domain SNP distribution

|  | **SNPs** *^a^* | | | | | **sSNPs** *^a^* | | | | | **nsSNPs** *^a^* | | | | |
| --- | --- | --- | --- | --- | --- | --- | --- | --- | --- | --- | --- | --- | --- | --- | --- |
| **PBP** | **Obs** | **Exp** | **Obs/Exp %** | **χ2** | **p** | **Obs** | **Exp** | **Obs/Exp %** | **χ2** | **p** | **Obs** | **Exp** | **Obs/Exp %** | **χ2** | **p** |
| *pbp1a* | 134 | 135 | 99.3 | 0.00 | 1.000 | 69 | 70 | 98.6 | 0.00 | 1.000 | 65 | 65 | 100.0 | 0.01 | 0.911 |
| *pbp1b* | 116 | 120 | 96.7 | 0.06 | 0.815 | 65 | 66 | 98.5 | 0.00 | 1.000 | 51 | 54 | 94.4 | 0.05 | 0.816 |
| *pbp2a* | 146 | 136 | 107.4 | 0.43 | 0.514 | 79 | 68 | 116.2 | 1.03 | 0.310 | 66 | 68 | 97.1 | 0.01 | 0.917 |
| *pbp2x* | 148 | 152 | 97.4 | 0.05 | 0.822 | 77 | 73 | 105.5 | 0.10 | 0.747 | 71 | 79 | 89.9 | 0.54 | 0.463 |

*^a^* Abbreviations: Obs = observed, Exp = expected.
