# Supplementary material for: Integrative Reverse Genetic Analysis Identifies Polymorphisms Contributing to Decreased Antimicrobial Agent Susceptibility in Streptococcus pyogenes
Source: mBio. 2022 Jan 18;13(1):e03618-21. doi: 10.1128/mbio.03618-21 (PMC8764543; doi:10.1128/mbio.03618-21)
Supplement: FIG S2 [file mbio.03618-21-sf002.pdf]

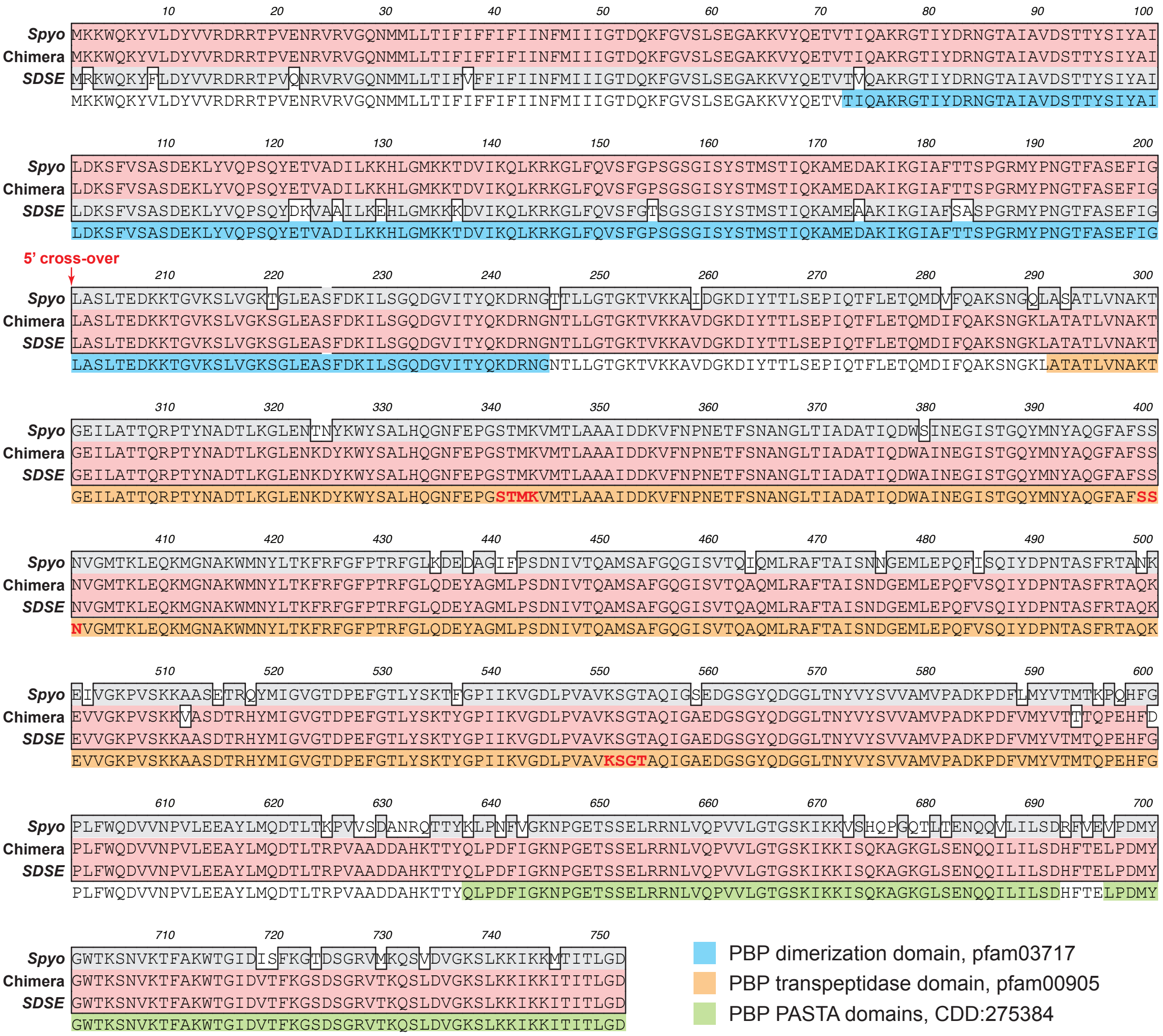

FIG S2 Aligned PBP2X sequences. Illustrated are sequences of the most prevalent *S. pyogenes* PBP2X variant (PBP2X-1), the chimeric variant (PBP2X-212), and PBP2X from SDSE strain NCTC7136 aligned with ClustalW. Regions of the alignment sharing identity are highlighted in red. Amino acids of the consensus sequence (below) are highlighted by functional domains as indicated in the inset lower right. The three transpeptidase key conserved catalytic motifs (SXXK, SXN, and KSGT) are shown in red.
