# Supplementary material for: Integrative Reverse Genetic Analysis Identifies Polymorphisms Contributing to Decreased Antimicrobial Agent Susceptibility in Streptococcus pyogenes
Source: mBio. 2022 Jan 18;13(1):e03618-21. doi: 10.1128/mbio.03618-21 (PMC8764543; doi:10.1128/mbio.03618-21)
Supplement: TABLE S8 [file mbio.03618-21-st008.docx]

**TABLE S8** PBP2X substitution isogenic construct primers

| **Substitution** | **PBP2X 5’ Amplicon Primers** | **PBP2X 3’ Amplicon Primers** | **pBBL740 Amplicon Primers** | **Strain** |
| --- | --- | --- | --- | --- |
| G_288_S | \| GTGAATACATCATGCATTTGTTGCCCAC \| \| --- \| \| TTCAAGCCAAATCAAATGGTCAGTTGGC \| | \| ACCATTTGATTTGGCTTGAAAAACATCC \| \| --- \| \| CAATTGTACATGCTAAACAGATTTTTCAAGC \| | \| CTGTTTAGCATGTACAATTGCTAGCGTAC \| \| --- \| \| CAAATGCATGATGTATTCACGAACGAAAATC \| | MGAS27213_  L_601_P-G_288_S |
| M_342_I | \| GTGAATACATTAGAACATCAATCCTCCAG \| \| --- \| \| GAGCCAGGCTCAACCATGAAAGTGATGAC \| | \| TTCATGGTTGAGCCTGGCTCAAAATTTC \| \| --- \| \| CAATTGTACAATGATGAATTGGGAGCGG \| | \| AATTCATCATTGTACAATTGCTAGCGTAC \| \| --- \| \| TGATGTTCTAATGTATTCACGAACGAAAATC \| | MGAS27213_  L_601_P-M_342_I |
| T_553_K | \| GTGAATACATCCTGCCATTCTGACCACTTC \| \| --- \| \| TGTTGCTGTTAAATCAGGAAAAGCACAAATTGG \| | \| TTCCTGATTTAACAGCAACAGGTAAATC \| \| --- \| \| CAATTGTACACATGATTATTATTGGAACAGATC \| | \| AATAATCATGTGTACAATTGCTAGCGTAC \| \| --- \| \| GAATGGCAGGATGTATTCACGAACGAAAATC \| | MGAS27213_  L_601_P-T_553_K |
| M_593_L | \| GTGAATACATATCAGTATTTGTGGGTCATC \| \| --- \| \| ACTTTTTGATGTATGTTACTTTGACTAAACC \| | \| AGTAACATACATCAAAAAGTCTGGTTTATCAG \| \| --- \| \| CAATTGTACAAAACCGTTACGATCCAAG \| | \| GTAACGGTTTTGTACAATTGCTAGCGTAC \| \| --- \| \| AAATACTGATATGTATTCACGAACGAAAATC \| | MGAS27213_  L_601_P-M_593_L |
| M_593_V | \| GTGAATACATATCAGTATTTGTGGGTCATC \| \| --- \| \| ACTTTTTGATGTATGTTACTGTGACTAAACC \| | \| AGTAACATACATCAAAAAGTCTGGTTTATCAG \| \| --- \| \| CAATTGTACAAAACCGTTACGATCCAAG \| | \| GTAACGGTTTTGTACAATTGCTAGCGTAC \| \| --- \| \| AAATACTGATATGTATTCACGAACGAAAATC \| | MGAS27213_  L_601_P-M_593_V |
| G_600_D | \| GTGAATACATATTCGCCAACTAAGTCAC \| \| --- \| \| CTAAACCACAACATTTTGATCCCCTTTTTTG \| | \| ATCAAAATGTTGTGGTTTAGTCATAGTAACATAC \| \| --- \| \| CAATTGTACAGCGATAGGAGAACTCCAG \| | \| CTCCTATCGCTGTACAATTGCTAGCGTAC \| \| --- \| \| GTTGGCGAATATGTATTCACGAACGAAAATC \| | MGAS27213_  L_601_P-G_600_D |
| P_601_H | \| GTGAATACATATTCGCCAACTAAGTCAC \| \| --- \| \| ACCACAACATTTTGGTCACCTTTTTTGG \| | \| GGTGACCAAAATGTTGTGGTTTAGTCATAGTAAC \| \| --- \| \| CAATTGTACAGCGATAGGAGAACTCCAG \| | \| CTCCTATCGCTGTACAATTGCTAGCGTAC \| \| --- \| \| GTTGGCGAATATGTATTCACGAACGAAAATC \| | MGAS27213_  L_601_P-P_601_H |
| P_601_S | \| GTGAATACATATTCGCCAACTAAGTCAC \| \| --- \| \| AAACCACAACATTTTGGTTCCCTTTTTTG \| | \| GAACCAAAATGTTGTGGTTTAGTCATAGTAAC \| \| --- \| \| CAATTGTACAGCGATAGGAGAACTCCAG \| | \| CTCCTATCGCTGTACAATTGCTAGCGTAC \| \| --- \| \| GTTGGCGAATATGTATTCACGAACGAAAATC \| | MGAS27213_  L_601_P-P_601_S |
